# Nanococrystals of Diclofenac Acid to Improve Biopharmaceutical Performance: Understanding the Key Drivers

**DOI:** 10.3390/pharmaceutics18091119

**Published:** 2026-09-06

**Authors:** Katangur Vishruth Reddy, Soumalya Chakraborty, Sourav Chougule, Amit Pariskar, Rohit Y. Sathe, Ashish Dangi, Prasad V. Bharatam, Arvind K. Bansal

**Affiliations:** 1 Department of Pharmaceutics, National Institute of Pharmaceutical Education and Research (NIPER), Sector-67, S.A.S. Nagar 160062, Punjab, India; vishruth.niper2014@gmail.com (K.V.R.);; 2 Department of Biological Sciences and Biotechnology, Institute of Chemical Technology, Matunga, Mumbai 400019, Maharashtra, India; 3 Department of Pharmacology and Toxicology, National Institute of Pharmaceutical Education and Research (NIPER), Sector-67, S.A.S. Nagar 160062, Punjab, India; 4 Department of Medicinal Chemistry, National Institute of Pharmaceutical Education and Research (NIPER), Sector-67, S.A.S. Nagar 160062, Punjab, India

**Keywords:** nanococrystals, cocrystals, diclofenac acid, dissolution, pharmacokinetics, microenvironmental pH, intermolecular interactions

## Abstract

**Background**: In this study, two cocrystals of diclofenac acid (DCA) with the coformers theophylline (THEO) and isonicotinamide (ISNT) were prepared. Subsequently, nanococrystals were generated from these cocrystals using a top-down wet media milling approach. **Methods**: Critical process parameters such as milling time, milling volume, drug loading percentage, bead volume, and dispersion media were optimized to achieve the desired particle size distribution. The nanococrystals were characterized using dynamic light scattering (DLS), polarized light microscopy (PLM), differential scanning calorimetry (DSC), powder X-ray diffraction (PXRD), Fourier transform infrared spectroscopy (FTIR), scanning electron microscopy (SEM), and transmission electron microscopy (TEM). **Results**: In vitro dissolution studies revealed that nanococrystals of DCA-ISNT (DE_0–120_ = 22.5% at pH 1.2 and DE_0–120_ = 58.7% at pH 4.5) and DCA-THEO (DE_0–120_ = 18.5% at pH 1.2 and DE_0–120_ = 48.2% at pH 4.5) exhibited superior dissolution performance compared to DCA nanocrystals (DE_0–120_ = 12.5% at pH 1.2 and DE_0–120_ = 39.0% at pH 4.5), with the dissolution advantage decreasing as the pH of the medium increased. The improved dissolution behaviour was a complex interplay of factors including particle size distribution, surface wetting kinetics, exposure of hydrophilic/hydrophobic functional groups during dissolution, nanococrystal microenvironmental pH, DCA’s ionization behaviour, lattice energy, and intermolecular interaction strengths. Additionally, nanococrystals exhibited a significantly higher flux rate in simultaneous gastric transfer dissolution and flux studies compared with DCA, likely due to higher apparent solubility and superior diffusion through the unstirred water layer (UWL). Pharmacokinetic studies confirmed that nanococrystals DCA-ISNT NCC (AUC_0–∞_ = 3062.65 ± 526.91 ng/mL·h) outperformed DCA nanocrystals (AUC_0–∞_ = 2352.53 ± 537.78 ng/mL·h), DCA-THEO NCC (AUC_0–∞_ = 2222.96 ± 151.19 ng/mL·h) and the cocrystals in terms of pharmacokinetic performance. **Conclusions**: The findings indicate that DCA-ISNT NCC exhibited superior pharmacokinetic performance and, together with the enhanced dissolution and flux properties of the nanococrystals, demonstrates their potential for enhanced therapeutic efficacy.

## 1. Introduction

Approximately 40% of approved drugs and nearly 90% of new chemical entities in the developmental pipeline are poorly water-soluble drugs (PWSDs) [1,2]. To address these low-solubility issues, various approaches have been developed. The commonly employed approaches to enhance the solubility of PWSDs include salt formation, amorphous solid dispersion, and lipidic systems [3,4]. The selection of an appropriate formulation strategy is guided by the physicochemical characteristics of the drug molecule and the desired biopharmaceutical outcome. Salt formation is a well-established strategy for improving the solubility of ionizable drug molecules [5]. However, non-ionizable drugs are not amenable to salt formation, and cocrystallization has emerged as a promising alternative for improving their solubility [6,7]. Pharmaceutical cocrystals consist of an API crystallized with one or more pharmaceutically acceptable coformers in a fixed stoichiometric ratio, where the components are held together within the same crystal lattice through noncovalent and non-ionic interactions [8].

Cocrystals have been reported to improve solubility by modulating crystal lattice interactions, microenvironmental pH, and generating supersaturation during dissolution [9,10,11]. However, cocrystallization does not always improve dissolution and may even reduce solubility or dissolution rate depending on the coformer and resulting crystal properties. Even when cocrystallization improves dissolution, the dissolution rate can still be limited by the particle size and surface properties of the resulting cocrystals [12,13]. Nanonization of cocrystals can provide an additional improvement by reducing particle size and increasing the available surface area, leading to the formation of nanococrystals [14,15].

Superior performance of nanococrystals in improving solubility and dissolution rates has been reported widely. However, the precise mechanisms by which nanococrystals achieve these improvements remain poorly understood [15,16,17]. Factors such as surface chemistry, crystallographic characteristics, particle size distribution (PSD), and microenvironmental pH are believed to play critical roles in nanococrystal performance [18]. Understanding these factors is critical for the rational development of nanococrystal formulations.

Diclofenac acid (DCA) is a weakly acidic non-steroidal anti-inflammatory drug (NSAID) classified under the Biopharmaceutical Classification System (BCS) as a Class II drug [19]. DCA is extensively used for the management of inflammatory disorders including rheumatoid arthritis, osteoarthritis, and gout because of its potent analgesic and anti-inflammatory activity [20,21]. However, its therapeutic efficacy is limited by poor solubility and dissolution, which ultimately affect its bioavailability [22]. This challenge underscores the need for advanced strategies to improve the solubility and dissolution behaviour of DCA, ultimately enhancing its absorption and therapeutic outcomes. Several formulation approaches have been investigated to improve the dissolution performance of DCA, including salt formation [23], self-emulsifying drug delivery systems [24], nanocrystals [25], nanocrystalline solid dispersions [22], and cocrystallization [26]. Salt formation has been investigated to improve the solubility and dissolution of DCA, while nanocrystals and nanocrystalline solid dispersions have been explored to enhance dissolution through particle size reduction and increased surface area. Cocrystallization has also been investigated as a strategy to modify crystal packing and improve the dissolution behaviour of DCA. However, the interplay between crystal properties and particle characteristics in determining the dissolution behaviour of these formulations is not yet fully understood.

The current study aims to generate nanococrystals of two reported cocrystals of DCA with theophylline (DCA-THEO, CSD refcode: OPOFUW) and isonicotinamide (DCA-ISNT, CSD refcode: UMUZAE) and investigate the key factors governing their enhanced dissolution performance. A deeper understanding of these parameters will offer mechanistic insights into the enhanced performance of nanococrystals, thereby guiding the development of more efficient drug delivery systems for poorly soluble drugs.

## 2. Methodology

### 2.1. Materials

Diclofenac acid (DCA, >99% purity) was obtained from JB Chemicals Pvt. Ltd. (Mumbai, India). The coformers (isonicotinamide and theophylline) and soya lecithin were procured from HiMedia Laboratories Pvt. Ltd. (Thane, India). Acetonitrile and n-dodecane were sourced from Sigma-Aldrich (St. Louis, MO, USA), while methanol was purchased from Rankem (New Delhi, India). Silicone oil was supplied by Loba Chemie Pvt. Ltd. (Mumbai, India), and orthophosphoric acid was purchased from Avra Synthesis Pvt. Ltd. (Hyderabad, India). All reagents and chemicals used in the work were of analytical grade.

### 2.2. Preparation of Cocrystals

Diclofenac acid cocrystals with theophylline (DCA-THEO) and isonicotinamide (DCA-ISNT) were prepared using the evaporative cocrystallization method. For DCA-THEO cocrystals, diclofenac and theophylline were dissolved in an equimolar ratio in methanol to obtain a clear solution. The solution was cooled, sealed with aluminum foil using Parafilm^®^(Bemis Company, Inc., Neenah, WI, USA), and pinholes were made on the foil to allow solvent evaporation at 40 °C. After complete evaporation, DCA-THEO cocrystals were collected and sieved through a British Standard Sieve (BSS) #36. Similarly, DCA-ISNT cocrystals were prepared by dissolving diclofenac and isonicotinamide in methanol to form a clear solution. The solution was sealed with aluminum foil using Parafilm^®^, with pinholes made for solvent evaporation at 40 °C. Upon complete evaporation, DCA-ISNT cocrystals were obtained and sieved through BSS #22.

### 2.3. Preparation of Nanococrystals and Nanocrystals

Nanocrystals DCA-NC of DCA as well as nanococrystals DCA-THEO NCC of DCA-THEO and DCA-ISNT NCC of DCA-ISNT were prepared via wet media milling. For each system, 150 mg (5% *w*/*v*) of DCA or the respective cocrystal was dispersed in 2.85 mL of aqueous medium containing 0.1% (*w*/*v*) SLS and 1% (*w*/*v*) HPC-EF as stabilizers. For DCA-THEO and DCA-ISNT, the aqueous medium was pre-saturated with the respective coformer. The dispersion was milled with 8.0 g of glass beads (0.3–0.4 mm) at 1500 rpm for 6 h. At regular intervals, 10 μL samples were withdrawn, diluted to 1 mL, and analyzed via dynamic light scattering (DLS). The resulting nanosuspensions were collected and stored at room temperature for further study.

### 2.4. Optimization of Wet Media Milling Process

The process parameters (amount of drug and stabilizer, stirring time and speed, size and amount of glass beads, and volume of the medium) were screened at different levels to investigate their effect on the media milling process. The description of process parameters is given in Table S1. The experimental design employed for estimating optimum values for each parameter, which would lead to nanocrystalline suspensions with least particle size and polydispersity index (PDI) to support its physical stability, is summarized in Table S2.

### 2.5. Solid-State Characterization

#### 2.5.1. Polarized Light Microscopy (PLM)

The powder samples DCA, DCA-THEO, DCA-ISNT, DCA-NC, DCA-THEO NCC, and DCA-ISNT NCC were observed using a Leica DMLP light microscope (Leica Microsystems Wetzlar GmbH, Wetzlar, Germany) in the polarized mode to detect birefringence in the samples. Microscopic images were captured using a JVS camera (Victor Company of Japan, Ltd., Yokohama, Japan) and Linksys32 software.

#### 2.5.2. Dynamic Light Scattering (DLS)

DLS analysis was performed with the help of Zetasizer Nano ZS (Nano ZS, Malvern Instruments, Worcestershire, UK). First, 100 μL media-milled dispersion was diluted to 10 mL using the saturated coformer solutions. Samples were then vortexed for up to 2 min for uniform dispersion. The prepared sample was transferred to the disposable cuvette. A backscatter detection angle of 173° was used to obtain Z_avg_ and PDI at 25 °C.

#### 2.5.3. Differential Scanning Calorimetry (DSC)

DSC thermograms of the investigated systems were recorded using DSC Q2000 (TA Instruments, New Castle, DE, USA) equipped with a refrigerated cooling accessory. The instrument was calibrated using indium prior to analysis. An accurately weighed sample (3–5 mg) was placed in an aluminum pan, equilibrated at 25 °C, and then heated at a rate of 10 °C/min up to 200 °C (for DCA, DCA-NC, DCA-ISNT, and DCA-ISNT NCC) and 290 °C (DCA-THEO and DCA-THEO NCC) with nitrogen gas purged at 50 mL/min. The recorded data were analyzed with Universal Analysis 2000 (version 4.5A).

#### 2.5.4. Powder X-Ray Diffraction (PXRD)

PXRD patterns of the samples were obtained using Rigaku Ultima IV diffractometer (Rigaku Corporation, Tokyo, Japan) equipped with Cu Kα radiation (λ = 1.5406 Å). The diffraction patterns were recorded over a 2θ range of 5–50° with a step size of 0.02° and a step time of 1 s.

#### 2.5.5. Scanning Electron Microscopy (SEM)

The morphology of the milled samples was observed using SEM (S-3400, Hitachi Ltd., Tokyo, Japan). Powder samples were mounted on double-sided adhesive tape placed on an aluminum stub for the analysis. The prepared samples were sputter coated using ion sputter (E-1010, Hitachi Ltd., Tokyo, Japan) at 10 Pa vacuum before analysis at 10 kV.

#### 2.5.6. Transmission Electron Microscopy (TEM)

Particle sizes of samples (DCA, DCA-NC, DCA-THEO, DCA-ISNT, DCA-THEO NCC, and DCA-ISNT NCC) were confirmed using TEM (FEI TF-20, Hillsboro, OR, USA). Powdered samples were dispersed in 1 mL of purified water and vortexed for 2 min to disperse the sample. A drop of this dispersion was placed on a copper grid and air-dried before analysis at 200 kV.

#### 2.5.7. X-Ray Photoelectron Spectroscopy (XPS)

XPS analysis was carried out using X-ray photoelectron spectrometer (Thermo Scientific, East Grinsted, UK) equipped with an Al Kα X-ray source and a Leybold-Heraeus hemispherical electron analyzer. Data were recorded in CAE mode with a pass energy of 50 eV, a 400 µm X-ray spot size, and an energy step of 0.1 eV.

### 2.6. Analytical Method for DCA Quantification

DCA concentrations were quantified using an HPLC system (LC-20AD, Shimadzu Corporation, Kyoto, Japan) equipped with a PDA detector. Separation was performed on a LiChrospher^®^ RP-C18 column (250 × 4.6 mm, 5 μm) using a mobile phase of 10 mM phosphate buffer (pH 3.0) and acetonitrile (25:75, *v*/*v*) at a flow rate of 1.0 mL/min. The injection volume was 20 μL, and detection was carried out at 280 nm.

### 2.7. In Vitro Dissolution Studies

The in vitro dissolution study was performed using the USP II dissolution apparatus. The HCl buffer (pH 1.2), acetate buffer (pH 4.5), and phosphate buffer (pH 6.8) were used as the dissolution medium. The 0.2% SLS was used in the dissolution medium as a surfactant. First, 900 mL of medium was taken in dissolution jars (*n* = 3) and the paddle speed of 50 rpm was maintained at 37 ± 0.2 °C. The drug and selected formulations of DCA-THEO, DCA-ISNT, DCA-THEO NCC, and DCA-ISNT NCC batches containing DCA equivalent to 35 mg were dispersed in dissolution release medium for dissolution testing. Samples were collected from the dissolution medium at 5, 15, 30, 45, 60, 90, and 120 min, filtered (0.1 μm nylon filters) and diluted for further analysis using HPLC. Dissolution efficiency (DE) over 0–30, 0–60, and 0–120 min was calculated from the mean dissolution profiles using the trapezoidal method described by Khan and Rhodes using the following formula [27].

$${DE}_{0-t}\left(\%\right)=\frac{\int_{0}^{t}Q(t)dt}{100\times t}$$

where *Q*(*t*) is the cumulative percentage of drug dissolved at time *t*, and *t* is the corresponding dissolution time (min).

### 2.8. Contact Angle Measurement

Contact angle experiments were performed using the sessile drop method with the help of a contact angle instrument (ACam-NSC, Apex Instruments, Kolkata, India). Samples were prepared by forming a powder layer using double-sided adhesive tape on the glass slide. Excessive powder was removed by tapping. A drop of liquid with a volume approximately 9–10 µL was dispensed and collected on the sample surface. The angle between the drop of liquid and the solid surface was measured using the tangent fitting method [28].

### 2.9. Microenvironmental pH and Microspecies Distribution

The microenvironmental pH of DCA, DCA-THEO, and DCA-ISNT was determined using the slurry method [29]. We acknowledge that measuring pH of the dissolving crystal surface is the most representative account of the microenvironment pH, and unlike that, equilibrium slurry pH does not capture influence of diffusion coefficients of drug and the buffer species [29,30]. However, many studies have proposed equilibrium slurry pH as a measurable proxy of microenvironmental pH [5,31,32,33,34,35]. The slurry was prepared by mixing 400 mg of the sample in 2 mL aqueous buffer (pH 1.2 and pH 4.5) with stirring for 30 min at 500 rpm. The pH of the slurry was measured using pH metre (Five Easy plus, Mettler Toledo, Greifensee, Switzerland) equipped with microelectrode. The microspecies distribution (%) of DCA, THEO and ISNT over the pH range of 0–7 was calculated using the Calculators & Predictors plugins integrated with JChem for Excel by ChemAxon (V. 16.11).

### 2.10. Lattice Energy Calculations

Total lattice energy (E_latt_) and its components were calculated using Growth Morphology module from Material Studio 2019 (Dassault Systems, BIOVIA, San Diego, CA, USA). The crystal structures of DCA (CSD refcode: SIKLIH05), DCA-THEO (CSD refcode: OPOFUW), and DCA-ISNT (CSD refcode: UMUZAE) were obtained from Cambridge Structural Database. The obtained structures were geometry optimized using *Forcite* module with COMPASS II force field. Geometry optimization was performed using the Smart algorithm with atomic positions optimized, an energy convergence tolerance of 1.0 × 10^−4^ kcal/mol, a force convergence criterion of 0.005 kcal/mol·Å, a displacement convergence criterion of 5 × 10^−5^ Å, and a maximum of 5000 optimization steps. Ewald summation was used for electrostatic interactions and an atom-based method was used for van der Waals interactions, with other parameters maintained at their default settings. Subsequently, the morphology of each crystal was predicted using the Growth Morphology module with the same force field. The calculation was performed using the Fine quality and Stable surfaces settings, with other parameters maintained at their default values. The calculation provided the predicted crystal faces, their relative surface areas, and corresponding attachment energies, including the total, van der Waals, and electrostatic contributions. The lattice energy and its components were obtained from the same calculation [36]. All Materials Studio calculations were performed using a valid institutional licence.

### 2.11. Analysis of Surface-Exposed Molecular Functionalities

Major crystal surfaces predicted during growth morphology calculation were analyzed for surface-exposed functionalities using the visualizer in the Material Studio 2019. The hydrophilic or hydrophobic nature of functional groups in the molecules was determined from molecular lipophilic surface mapping. MLSP mapping of DCA, THEO, and ISNT was performed with VEGA ZZ software (version 3.2.3, V3.2.3, Drug Design Laboratory, University of Milan, Milan, Italy) using the virtual logP algorithm of Bernard Testa et al. and probe radius of 1.4 Å [37,38].

### 2.12. Energy Framework Analysis

Intermolecular interaction energies between individual molecular pairs in the crystal structure were calculated using CrystalExplorer 21.5 software. Pairwise interaction energies along with contribution from electrostatic and dispersive components were determined using CE-B3LYP/6-31G(d,p) electron density model. The calculated energies were used to generate energy framework diagram with energy cut-off of 5 kJ/mol [39].

### 2.13. Simultaneous Dissolution and Flux Experiments

#### 2.13.1. Preparation of Artificial Membrane

A membrane-forming solution was prepared by dissolving 20% (*w*/*v*) soya lecithin in n-dodecane containing 1.5% (*v*/*v*) absolute ethanol. For this, soya lecithin was added in the mixture of ethanol and n-dodecane gradually and stirred overnight (~12 h at 500 rpm) at ambient temperature. Thereafter, the solution was centrifuged at 3220× *g* at 20 °C for 20 min to remove any undissolved material. The supernatant was collected and stored at −20 °C until further use [40]. Before use, the membrane-forming solution was thawed overnight at room temperature. Artificial membranes were prepared by applying 16.2 µL/cm^2^ of the membrane-forming solution onto a PVDF filter support (0.45 µm pore size) and allowing it to equilibrate for 10 min before use.

#### 2.13.2. Simultaneous Dissolution-Flux Experiments

Samples (DCA, DCA-NC, DCA-THEO, DCA-ISNT, DCA-THEO NCC, and DCA-ISNT NCC) were tested using an in-house apparatus. The receiver compartment integrated with the artificial membrane and an overhead stirrer was positioned inside a 1000 mL glass beaker. The artificial membrane separated the receiver and donor compartments. Then 10 mL of pH 6.8 phosphate buffer containing 2% (*w*/*v*) SLS (sink medium) was added to the receiver compartment. The representative diagram of the apparatus is shown in Figure S1. The experiment was initiated with 300 mL of pH 1.2 buffer in the donor compartment to simulate gastric conditions. After 15 min, 600 mL of concentrated pH 6.8 phosphate buffer was added to the donor medium to simulate intestinal conditions (pH 6.8). Stirring rates of 100 rpm and 250 rpm were maintained in the donor and receiver compartments, respectively, to minimize the thickness of unstirred water layer [41]. The aliquots were collected from the donor compartment at 5, 10, 15, 20, 25, 30, 45, 75, 105, and 135 min, and from the receiver compartment at 20, 25, 30, 45, 75, 105, and 135 min. Drug concentrations were quantified using HPLC. The flux across the membrane was calculated between 15 and 135 min using the following equation [42,43].

(1) $$J=\frac{m}{A∆t}$$

where *J* is the flux of a drug across membrane, *m* is amount of drug crossing, *A* is area, and t is a time.

### 2.14. Bioanalytical Method for Quantification of DCA in Rat Plasma

Diclofenac acid concentrations in plasma were quantified using an HPLC system (LC-20AT, Shimadzu, Kyoto, Japan) equipped with a PDA detector. Chromatographic separation was performed on a LiChrospher^®^ 100 RP-C18 column (250 × 4.6 mm, 5 μm; Merck KGaA, Darmstadt, Germany). The mobile phase consisted of ACN and 10 mM phosphate buffer (pH 3.0) (65:35, *v*/*v*) with the flow rate of 1.0 mL/min. The injection volume was 90 μL, and detection was carried out at 280 nm. Naproxen was used as the internal standard.

### 2.15. Pharmacokinetic Study

The pharmacokinetic evaluation was conducted in male Sprague-Dawley rats weighing 180–200 g following approval from the Institutional Animal Ethics Committee (Protocol No. IAEC/21/01/Ext-1). Animals were acclimatized for one week under controlled housing conditions with a 12 h light/12 h dark cycle. Fresh suspensions of DCA, DCA-NC, DCA-THEO, DCA-ISNT, DCA-THEO NCC, and DCA-ISNT NCC were prepared in sodium carboxymethyl cellulose (0.5% *w*/*v*) to provide an oral dose equivalent to 3 mg/kg. Prior to dosing, the rats were fasted overnight (12 h) while allowing free access to water. Each treatment group consisted of six male Sprague-Dawley rats, which received the formulations by oral gavage at a dose equivalent to 3 mg/kg of diclofenac acid and a dosing volume of 2 mL/kg. Blood samples were collected from the tail vein at 0, 0.167, 0.25, 0.5, 0.75, 1, 2, 4, and 6 h after oral administration. Samples were transferred into K2-EDTA-containing tubes, and maintained on ice until they were centrifuged at 7000 rpm for 10 min at 4 °C to separate the plasma. The harvested plasma was stored at −80 °C until analysis. Before sample preparation, frozen plasma samples were allowed to thaw at room temperature. A 100 µL plasma aliquot was mixed with 10 µL of the internal standard solution (naproxen, 150 µg/mL in the mobile phase) in a microcentrifuge tube and vortexed for 2 min. Protein precipitation was performed by adding 190 µL of an ACN:methanol mixture, followed by vortex mixing for 5 min. The mixture was then centrifuged at 10,000 rpm for 10 min at 4 °C. Further, 150 µL of the clear supernatant was collected and analyzed using HPLC.

### 2.16. Statistical Analysis

The PK parameters of DCA and its formulations were determined using PK Solver. The experimental data are expressed as mean ± SD. Non-compartmental analysis was employed to estimate C_max_, T_max_, and AUC_0–∞_. Data were analyzed using one-way ANOVA followed by Tukey’s multiple-comparisons test. Differences were considered statistically significant at *p* < 0.05.

## 3. Results and Discussion

### 3.1. Solid-State Characterization

**Figure 1:**
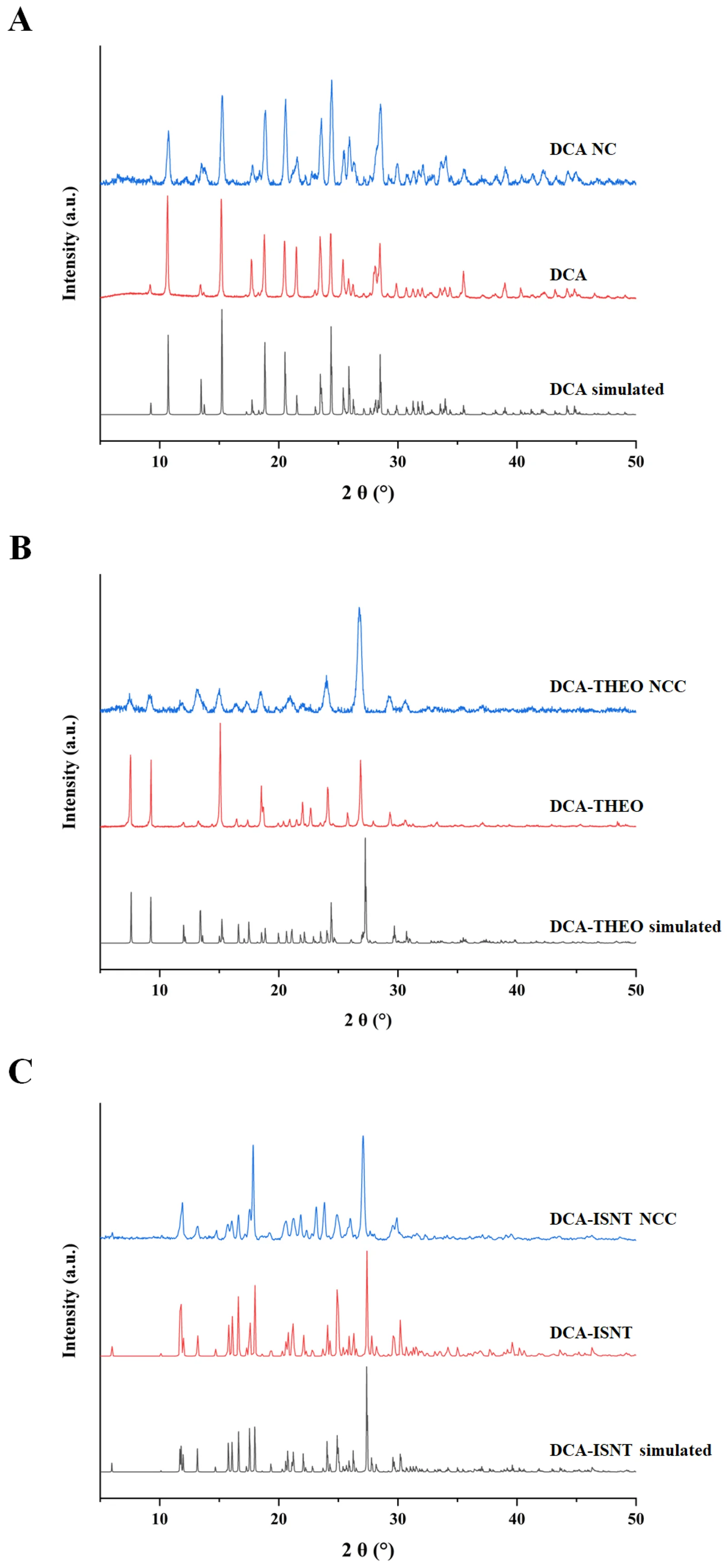
PXRD overlay of simulated before and after milling patterns of (**A**) DCA, (**B**) DCA-THEO, and (**C**) DCA-ISNT, confirming retention of crystalline form. The simulated patterns for DCA, DCA-THEO, and DCA-ISNT were generated from the reported crystal structures (CSD refcode: SIKLIH05, OPOFUW and UMUZAE, respectively).

Solid-state characteristics of diclofenac acid (DCA), theophylline (THEO), and isonicotinamide (ISNT) were investigated to establish their crystalline nature and for verification of subsequent solid forms. Their crystalline nature was evident from birefringence under PLM (Figure S2) and DSC. DCA exhibited a melting point at 178.4 °C with enthalpy of fusion of 124.7 J/g, while THEO and ISNT showed melting points of 271.0 °C and 156.9 °C with enthalpies of fusion of 162.0 J/g and 194.2 J/g, respectively (Figure S3). Similarly, both DCA-THEO and DCA-ISNT cocrystals exhibited strong birefringence under PLM (Figure S4), confirming their crystalline nature. DCA-THEO exhibited a sharp melting endotherm at 187.6 °C with enthalpy of fusion of 102.4 J/g, in excellent agreement with the previously reported melting point of 186.9 °C by Surov et al. [44]. DCA-ISNT exhibited a melting endotherm at 166.3 °C with an enthalpy of fusion of 135.4 J/g, which is in close agreement with the previously reported melting point of 164.0 °C by Bathori et al. (Figure S5) [45]. The PXRD analysis further confirmed the formation of these cocrystals as the experimental patterns matched the simulated patterns obtained from the reported crystal structure (Figure 1) [44,45].

After wet media milling, the DCA nanocrystal system (DCA-NC) maintained its crystalline structure, as evidenced by PXRD (Figure 1). A slight reduction was observed in melting point (177.98 °C) and enthalpy of fusion (105.4 J/g) due to particle size reduction. Similarly, the nanococrystals of both systems (DCA-THEO NCC and DCA-ISNT NCC) maintained their crystalline integrity, with slight reductions in melting points. DCA-THEO NCC showed a melting point of 185.29 °C, while DCA-ISNT NCC exhibited a melting point of 164.09 °C (Figure S6). PXRD analysis of the milled samples confirmed that no phase transitions occurred during milling (Figure 1).

**Figure 2:**
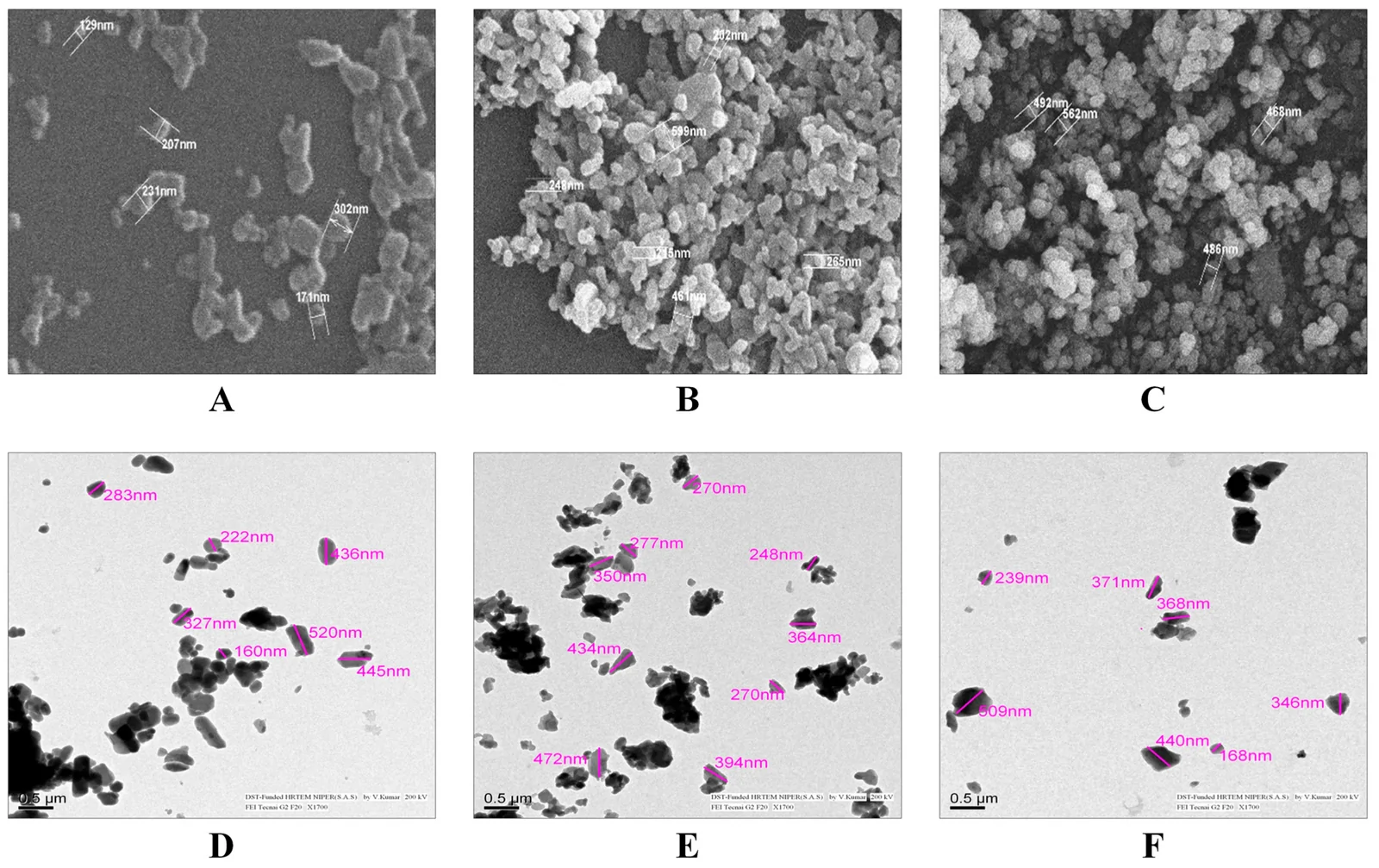
Representative SEM (**A**–**C**) and TEM (**D**–**F**) micrographs of DCA nanocrystals (**A**,**D**), DCA-THEO NCC (**B**,**E**), and DCA-ISNT NCC (**C**,**F**).

SEM and TEM indicated nanoscale particle sizes for both nanococrystal systems, confirming effective size reduction as shown in Figure 2. The micrographs showed irregular to rounded particle morphologies, with some variation in particle size and shape among the three systems. Particle clustering was also observed in densely packed regions. Particle size distribution (PSD) analysis using DLS (Table S3) further supported the nanonization of particles. Both nanocrystals exhibited monomodal particle size distributions. The differences in PDI may arise from particle aggregation and/or the coexistence of different particle size populations of non-aggregated crystallites within a narrow range. These contributions cannot be reliably deconvoluted by DLS alone. Fourier transform infrared (FTIR) spectroscopy showed no new absorption bands or significant shifts in the spectra of milled samples, confirming the absence of degradation during the milling process (Figure S7). Overall, the solid-state characterization confirmed the crystalline stability and integrity of the cocrystal and nanococrystal systems.

### 3.2. In Vitro Dissolution Study

**Figure 3:**
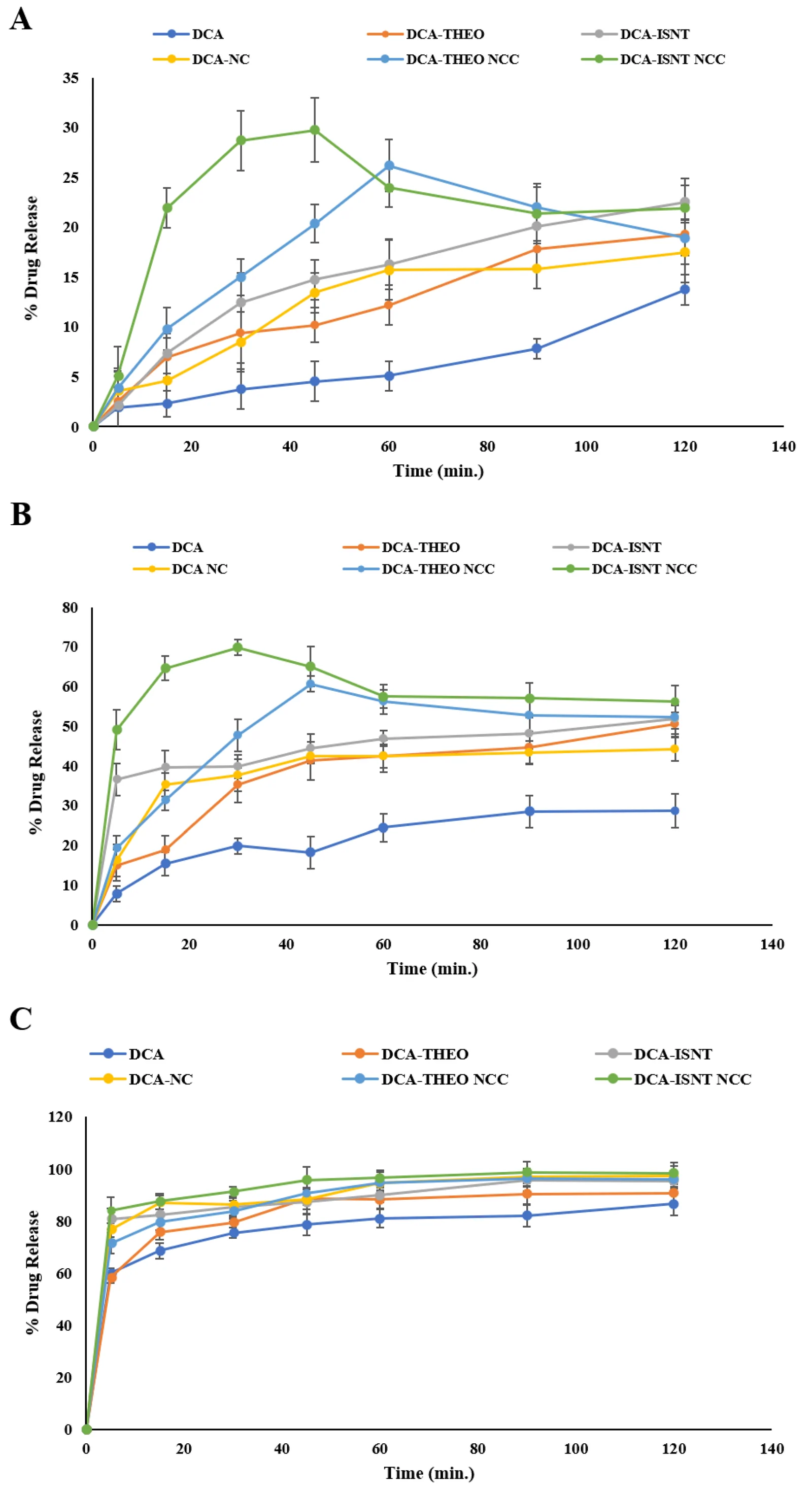
Dissolution profile of the studied systems (n = 3) in (**A**) pH 1.2 buffer + 0.2% SLS, (**B**) pH 4.5 buffer + 0.2% SLS, (**C**) pH 6.8 buffer + 0.2% SLS.

The influence of cocrystallization and subsequent nanonization on drug dissolution was investigated under three physiologically relevant dissolution conditions, where pH 1.2, 4.5, and 6.8 represent the gastric, jejunal, and duodenal pH, respectively. The dissolution behaviour of the investigated systems is shown in Figure 3, while the dissolution efficiencies (DEs) calculated up to 30, 60, and 120 min are summarized in Table 1. DE provides a quantitative measure for comparing dissolution performances by integrating both the rate and extent of drug release over a defined time period.

At pH 1.2, pronounced differences were observed in the dissolution behaviour of investigated systems. DCA exhibited the lowest dissolution efficiency throughout the study, with DE values of 2.4%, 3.4%, and 6.0% at 30, 60, and 120 min, respectively. DCA, a weakly acidic drug, has a pKa of 4.01 due to ionization of the hydroxyl functionality of the carboxyl moiety. At pH 1.2, it is predominantly in unionized form, thus reducing its solubility in water. Cocrystallization significantly enhanced the dissolution performance of DCA, increasing the DE_0–120_ to 12.5% for DCA-THEO and 15.2% for DCA-ISNT. Nanonization further amplified this improvement, as evident from DE_0–120_ values of 12.5%, 18.5%, and 22.5% for DCA-NC, DCA-THEO NCC, and DCA-ISNT NCC, respectively. The effect of nanonization was more significant during the initial stage of dissolution, where DCA-ISNT NCC exhibited DE_0–30_ of 17.6%, which was approximately 2.0-fold higher than DCA-THEO NCC (8.8%), 2.6-fold higher than DCA-ISNT (6.7%), and 7.3-fold higher than DCA (2.4%). However, DCA-ISNT NCC showed only marginal change in DE between 60 and 120 min, consistent with the dissolution profile, which showed a slight decrease in dissolved drug concentration after reaching the maximum. This behaviour may be attributed to the relaxation of transient supersaturation and crystallization.

At pH 4.5, all samples exhibited higher dissolution efficiencies than at pH 1.2, possibly due to the increased ionization and apparent solubility of DCA near its pKa. The overall dissolution trend remained the same, with DCA (22.1%) and DCA-ISNT NCC (58.7%) showing the lowest and highest DE_0–120_, respectively. Although the dissolution trend was similar, the differences among the studied systems were less pronounced than those observed at pH 1.2. DCA-ISNT NCC exhibited the highest initial dissolution efficiency (DE_0–30_ = 56.6%), which was approximately 1.5-fold higher than DCA-ISNT (35.6%) and 4.25-fold higher than DCA (13.3%). Similarly to pH 1.2, DCA-ISNT NCC showed minimal change in DE between 60 and 120 min (60.5% and 58.7%, respectively), which is consistent with the slight decrease in dissolved drug concentration.

At pH 6.8, all samples exhibited rapid and higher dissolution with fewer differences in dissolution efficiency than those observed at pH 1.2 and pH 4.5. The DE_0–120_ increased from 76.7% for DCA to 83.0% for DCA-THEO, 87.9% for DCA-ISNT, 89.8% for DCA-NC, 88.5% for DCA-THEO NCC, and 92.8% for DCA-ISNT NCC. The initial dissolution efficiencies (DE_0–30_) ranging from 62.5% for DCA to 80.4% for DCA-ISNT NCC indicate that all systems dissolved rapidly under sink conditions at pH 6.8. Unlike at pH 1.2 and 4.5, the decrease in dissolved drug concentration after reaching the maximum in the case of nanococrystals was not observed at pH 6.8. The reduced discrimination in the dissolution profile of samples can be attributed to the highest percent of ionized species of DCA at pH significantly above its pKa. This markedly increases its apparent solubility and reduces the influence of cocrystallization and nanonization on dissolution performance. However, DCA-ISNT NCC consistently exhibited the highest dissolution efficiency throughout the study, indicating that the beneficial effects of cocrystallization and nanonization were retained even under highly solubilizing conditions.

To quantitatively compare the effects of cocrystallization and subsequent nanonization, the relative increase in DE_0–120_ was calculated for each formulation step. At pH 1.2, cocrystallization increased DE_0–120_ by 108.3% for DCA-THEO and 153.3% for DCA-ISNT. Subsequent nanonization produced a further 48.0% increase for both systems. At pH 4.5, cocrystallization increased DE_0–120_ by 71.0% and 100.5% for DCA-THEO and DCA-ISNT, respectively. Nanonization further increased DE_0–120_ by 27.5% and 32.5%, respectively. At pH 6.8, the increases were smaller. Cocrystallization increased DE_0–120_ by 8.2% for DCA-THEO and 14.6% for DCA-ISNT, while nanonization produced further increases of 6.6% and 5.6%, respectively. These comparisons indicate that both cocrystallization and nanonization contributed to the improved dissolution, although their relative effects varied with pH and cocrystal system.

### 3.3. Mechanistic Understanding of Dissolution Behaviour

The dissolution behaviour of DCA, DCA-THEO, DCA-ISNT cocrystals and their nanosized counterparts is governed by the complex interplay of multiple factors such as their PSD, lattice energy, surface molecular environment of crystal facets, state of ionization of DCA as a function of pH, and microenvironmental pH of SWL around dissolving particles.

#### 3.3.1. Particle Size Distribution

The unmilled DCA, DCA-THEO, and DCA-ISNT exhibited D_90_ values of 81.5, 85.0, and 87.0 µm, respectively, under microscopic evaluation, whereas the media-milled samples exhibited submicron particle sizes. The Z_avg_ of milled samples measured by the DLS are given in Table S3. While DLS measures the hydrodynamic size of particles, SEM and TEM images demonstrated submicron-sized particles in range of 200–500 nm (Figure 1). All the nanosized samples exhibited higher dissolution efficiency than their unmilled counterparts. However, the difference in extent of dissolution improvement due to nanonization suggests that the particle size reduction alone is insufficient to explain this behaviour.

#### 3.3.2. Surface Wettability and Crystal Surface Chemistry

**Figure 4:**
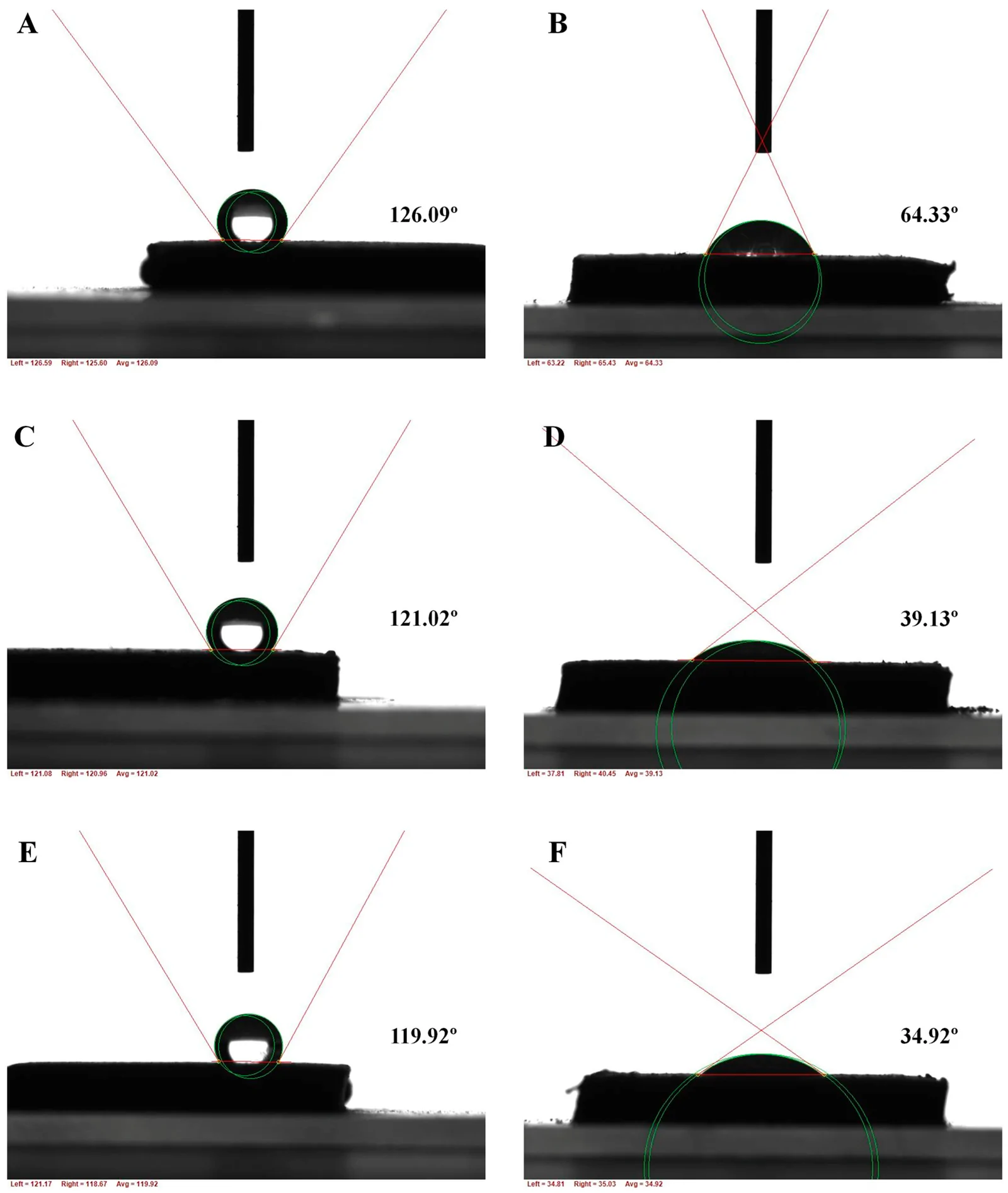
Contact angle of water on the surface of (**A**) DCA, (**B**) DCA-NC, (**C**) DCA-THEO, (**D**) DCA-THEO NCC, (**E**) DCA-ISNT, and (**F**) DCA-ISNT NCC.

The initial interaction of the dissolution medium with the particle is governed by the surface wettability of the solid. The sessile drop contact angle method was used to capture the surface wettability of milled and unmilled samples. The contact angle of deionized water drop on the surface of milled and unmilled samples is demonstrated in Figure 4. Cocrystals showed a slightly lower contact angle (121.02° for DCA-THEO and 119.92° for DCA-ISNT) compared to that of DCA (126.09°), whereas all the nanonized samples exhibited a significant reduction in contact angle. The marked decline in the contact angle indicates the improved wettability of the DCA-NC (64.33°), DCA-THEO NCC (39.13°), and DCA-ISNT NCC (34.92°). The observed differences in wettability are likely associated with differences in the molecular environment of the exposed crystal surfaces [46].

The surface elemental compositions of investigated systems determined by XPS are summarized in Table S4. Both nanococrystals exhibited a slight reduction in surface carbon content accompanied by an increase in oxygen content compared with unmilled samples. Although XPS provides quantitative information on the elemental composition of the outermost ~5–10 nm of particle surface, it does not distinguish the chemical nature of the exposed functional groups. These observed differences in elemental composition alone cannot adequately explain the differences in wettability and dissolution behaviour. Therefore, the surface chemistry of the dominant crystal facets was further investigated to identify the hydrophilic functionalities exposed at the major crystal surfaces (Figure S9). The experimentally observed crystal habits of the bulk cocrystals obtained by solvent evaporation showed qualitative consistency with the corresponding predicted growth morphologies (Figure S8). The assignment of the exposed functionalities as hydrophilic or hydrophobic was further supported by the MLSP maps (Figure S10).

The dominant facets of DCA were largely devoid of surface-exposed hydrophilic functionalities, except the (1 1 0) facet exposing a carboxylic acid group (Table 2). In contrast, the major facets of both cocrystals presented additional hydrogen-bonding functionalities capable of interacting with water. DCA-THEO predominantly exposed lactam carbonyl and secondary amine functionalities, whereas DCA-ISNT exposed amide, carboxylic acid, and pyridine nitrogen functionalities, which provide a more complementary distribution of hydrogen-bond donor and acceptor sites at the crystal surface. This favourable surface chemistry is consistent with the improved wettability observed for both cocrystals. These surface characteristics may also contribute to the enhanced initial dissolution after nanonization, with DCA-ISNT NCC exhibiting the most pronounced effect.

The relevance of the facet-specific surface chemistry to the milled systems was further evaluated through cleavage-plane analysis. The preferred cleavage plane was assigned based on the combined consideration of visualization, d-spacing (*dhkl*), and attachment energy (Table S5). In all three systems, the most dominant facet exhibited the largest d-spacing along with the least negative attachment energy. Additionally, visualization of supercell structures using Mercury software revealed the absence of H-bonding contacts in these planes (Figure S11). These structural features suggest that fracture during wet media milling may preferentially occur along the dominant crystal facet. Therefore, milling may generate surfaces with surface chemistry similar to that of the dominant facet. However, the specific facet exposure of the resulting nanococrystals was not experimentally confirmed. Thus, this relationship is proposed as a structural mechanism and adequately explains the experimental observations. It should also be noted that SLS and HPC-EF, used during nanococrystal preparation, may contribute to the observed surface wettability and dissolution behaviour. However, the same stabilizer composition and milling conditions were used for DCA-NC and both nanococrystal systems. Their potential contribution cannot be completely excluded.

#### 3.3.3. Microenvironmental pH and Microspecies

**Figure 5:**
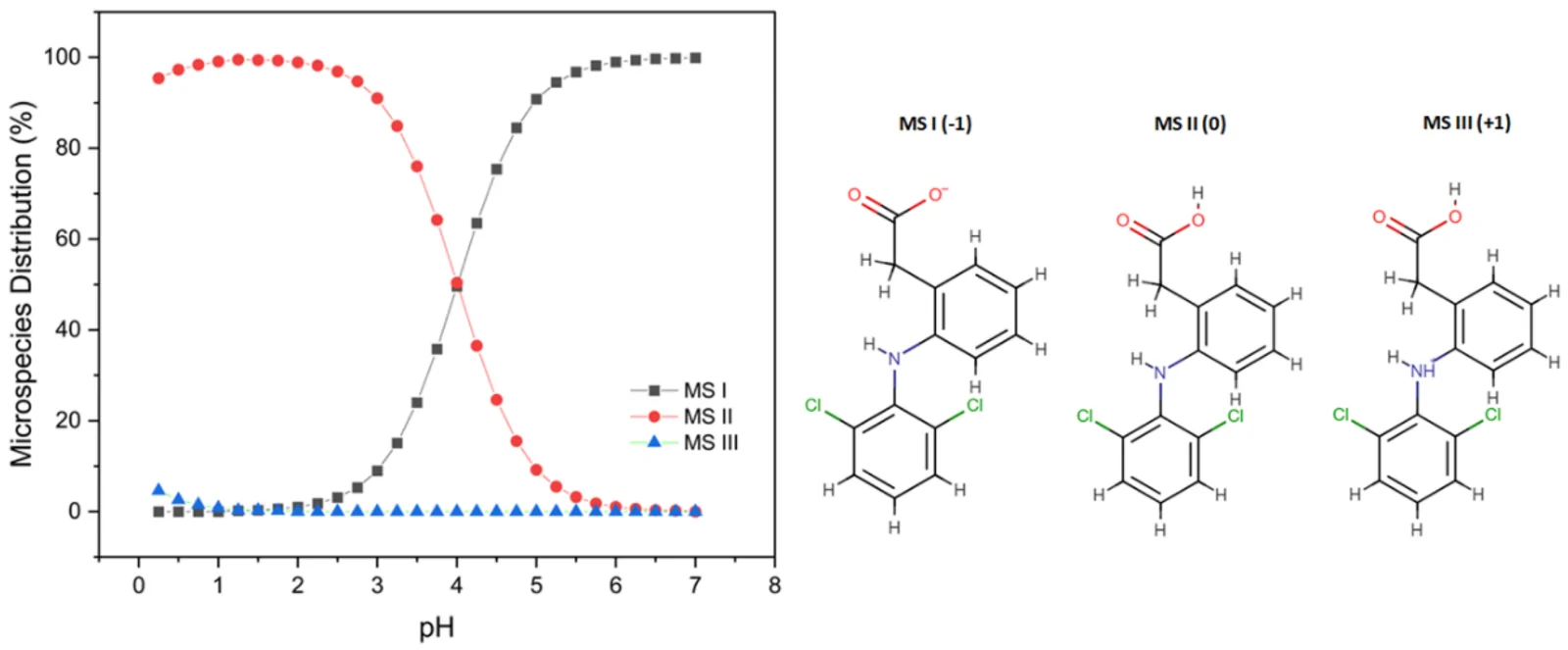
Percentage microspecies distribution of DCA over the pH range of 0–7.

The pH of the dissolution medium plays a very important role in regulating the dissolution performance of APIs with pH-dependent solubility. DCA is a weakly acidic compound with a pKa of 4.01. Thus, it demonstrates relatively lower ionization tendency at acidic pH and higher ionization propensity at higher pH, according to the Henderson–Hasselbalch Equation [46]. The pH-dependent ionization and microspeciation tendency of DCA lead to three microspecies (MS I-MS III) over the pH range of 0–7 (Figure 5). It is evident that at pH values below pKa, the percentage distribution of unionized MS II is higher. Thus, DCA exhibits higher solubility at pH values higher than the pKa value. Overall, the dissolved drug concentration is significantly higher in pH 6.8 compared to pH 1.2 and pH 4.5.

It has been recognized that the dissolution is governed primarily by the conditions within the unstirred water layer (UWL) adjacent to the particle surface rather than by the bulk dissolution medium [47]. The pH within this localized region is referred to as the microenvironmental pH. It represents the hydrogen ion activity within the UWL, where mass transport occurs primarily by diffusion due to negligible convective mixing. Therefore, changes in the microenvironmental pH can significantly influence dissolution rate, solubility, and oral bioavailability for the drugs with pH-dependent solubility [29]. Various studies have established that modulation of the microenvironmental pH plays a key role in the dissolution performance of salts [35], cocrystals [47], and eutectics [48].

Table 3 represents the microenvironmental pH of DCA, DCA-THEO, and DCA-ISNT at pH 1.2 and pH 4.5 aqueous buffers.

The measured microenvironmental pH values demonstrated distinct behaviour for the two cocrystals. DCA-THEO increased the microenvironmental pH only under the strongly acidic condition (pH 1.2), whereas only a marginal change was observed at pH 4.5. This behaviour is consistent with the pH-dependent microspecies distribution of THEO (Figure S12). At pH 1.2, the protonated microspecies (MS III, +1) predominates, which facilitates proton uptake, increasing the local pH. However, the neutral microspecies (MS II) dominates between pH 3–7. Thus, no change in pH was observed. In contrast, DCA-ISNT raised the microenvironmental pH at both pH 1.2 and 4.5, with a significantly greater increase at pH 1.2 (3.81) than at pH 4.5 (4.91). This behaviour is attributed to the pH-dependent protonation of the pyridine nitrogen of ISNT (Figure S13). The protonated microspecies predominates at pH 1.2 (99.4%) but decreases considerably at pH 4.5 (8.4%). Thus, the ability of ISNT to modify the local dissolution environment is greatest under strongly acidic conditions.

The elevation of the microenvironmental pH shifts its ionization equilibrium towards the ionized species, thereby increasing its apparent solubility within the UWL. The percentage of unionized DCA (MS II) was consistently lower for DCA-ISNT than for DCA, whereas DCA-THEO exhibited only a marginal change in DCA microspeciation (Table 3). This modulation of the microenvironmental pH was reflected in the dissolution behaviour under acidic conditions. At pH 1.2, DCA-ISNT exhibited higher DE_0–120_ than DCA-THEO (15.2% vs. 12.5%), with a more pronounced difference for the corresponding nanococrystals (22.5% vs. 18.5%). A similar trend was observed at pH 4.5, where DCA-ISNT and DCA-ISNT NCC showed higher DE_0–120_ values than DCA-THEO and DCA-THEO NCC. These findings indicate that modulation of the microenvironmental pH provides an additional dissolution advantage to the ISNT system, particularly under non-sink conditions.

#### 3.3.4. Crystal Lattice Energy and EFA

Lattice energy (E_latt_) is the energy required to separate one mole of a crystalline solid into isolated gaseous molecules. It reflects the overall strength of intermolecular interactions stabilizing the crystal lattice. During dissolution, these intermolecular interactions must be overcome to enable molecular detachment from the crystal surface [49]. The calculated lattice energies and the contributions of individual interaction components are summarized in Table 4. Cocrystallization significantly reduced the lattice energy from −283.9 kcal/mol for DCA to −135.8 and −126.1 kcal/mol for DCA-THEO and DCA-ISNT, respectively. The decrease in E_latt_ was primarily attributed to the marked reduction in vdW interactions. DCA-ISNT exhibited the least negative lattice energy, suggesting the lowest energetic barrier for molecular detachment from the crystal lattice, which is consistent with its superior dissolution behaviour.

To gain molecular-level insight into the origin of the reduced lattice energy, the intermolecular interaction energies between neighbouring molecular pairs were analyzed. While lattice energy provides an overall measure of crystal stability, pairwise interaction analysis identifies the dominant intermolecular interactions contributing to crystal packing. This offers insight into the interactions that must be disrupted for molecular detachment during dissolution. The strongest intermolecular interaction energies are summarized in Table 5. The complete interaction energy calculations and corresponding energy framework diagrams are provided in the Supplementary Information (Tables S6–S8 and Figures S14–S16). DCA exhibited a strong DCA···DCA interaction with a total interaction energy (E_tot_) of −73.4 kJ/mol, reflecting the tightly packed crystal structure. In contrast, dominant intermolecular interactions were significantly weakened in the case of cocrystals. The strongest interaction in DCA-THEO (between DCA···THEO molecular pair) showed an E_tot_ of −30.9 kJ/mol, while the strongest interactions in DCA-ISNT (between DCA···ISNT molecular pair) exhibited an E_tot_ of only −20.0 kJ/mol. These results suggest that cocrystallization replaces the strong homomolecular DCA···DCA interactions with weaker heteromolecular interactions, which may facilitate molecular detachment during dissolution. The comparatively weaker dominant interactions in DCA-ISNT are consistent with its lower lattice energy. This suggests a crystal packing arrangement that may facilitate molecular detachment from the crystal lattice during dissolution.

Collectively, these findings suggest that the enhanced dissolution behaviour of the nanococrystals arises from the interplay of particle engineering and crystal engineering rather than particle size reduction alone. While nanonization improved the dissolution of all milled systems, the superior dissolution efficiency of DCA-ISNT NCC could not be explained solely by particle size. DCA-ISNT NCC combined improved wettability, favourable microenvironmental pH, and reduced lattice stability, which may collectively contribute to enhanced molecular detachment and dissolution. The combined contribution of these factors is consistent with the higher dissolution efficiency of DCA-ISNT NCC compared with DCA-THEO NCC and DCA-NC across the investigated dissolution media.

### 3.4. Simultaneous Gastric Transfer Dissolution and Flux Study

**Figure 6:**
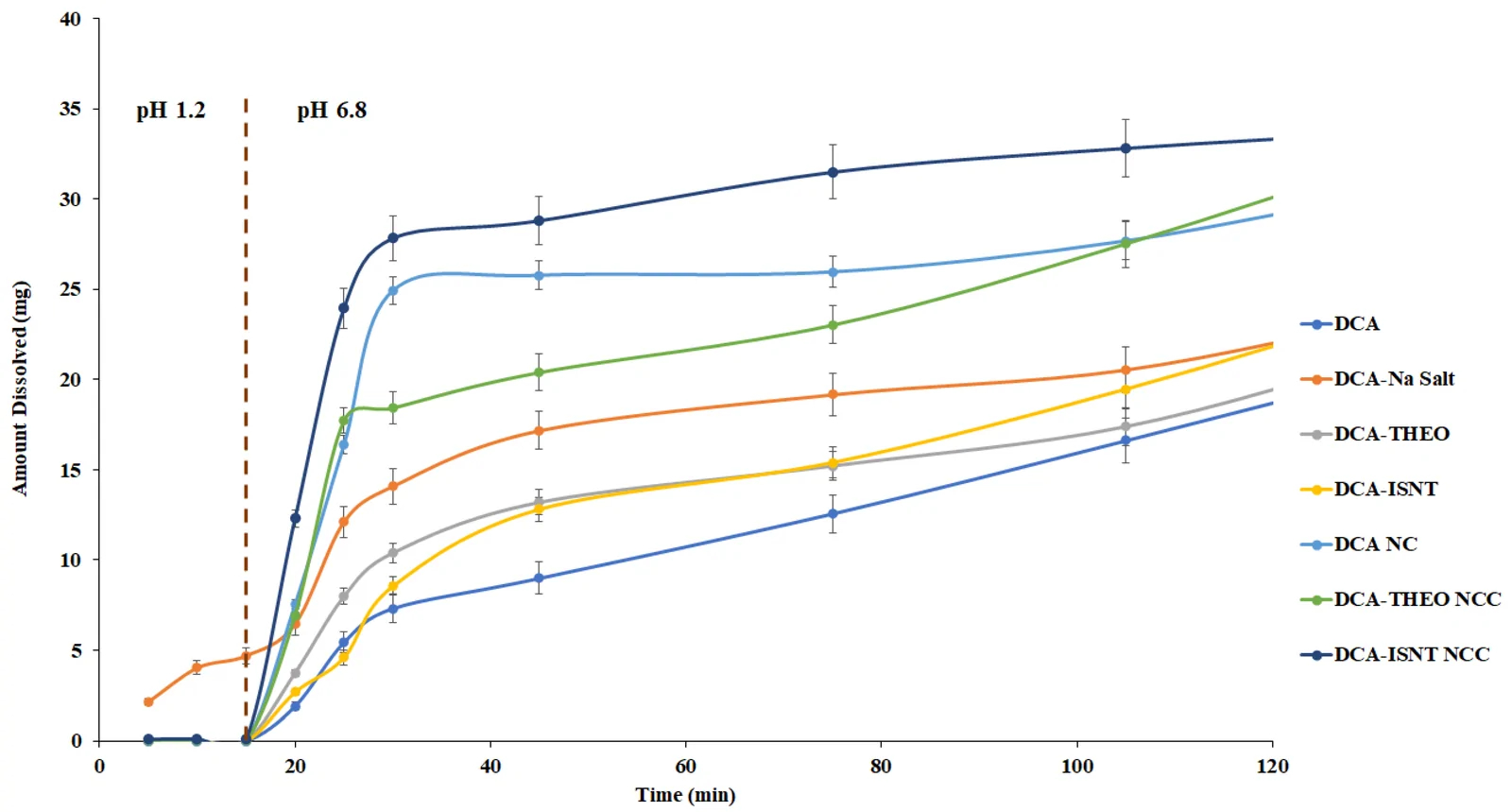
Dissolution profile of the studied materials in pH 1.2 and pH 6.8 in gastric transfer model (n = 3). The vertical dash line represents the transition from pH 1.2 to pH 6.8.

The gastric transfer dissolution model was employed to evaluate the dissolution behaviour of the investigated systems along with DCA-Na salt in sequential gastric-intestinal conditions. This simulates the physiological pH transition following oral administration. Due to the negligible solubility of DCA under gastric conditions, the gastric transfer model included DCA-Na salt as an additional reference representing the conventional salt form of diclofenac. Negligible drug release was observed for DCA, DCA-NC and the unmilled cocrystals during the gastric phase (pH 1.2), whereas DCA-THEO NCC (0.047 mg) and DCA-ISNT NCC (0.106 mg) showed detectable drug release (Figure 6). In contrast, DCA-Na salt showed rapid dissolution with 4.71 mg of DCA released within 15 min. Following transfer to the intestinal medium (pH 6.8), dissolution increased noticeably for all formulations due to the higher solubility of DCA at intestinal pH. However, DCA-ISNT NCC consistently exhibited the highest drug release, followed by DCA-THEO NCC and DCA-NC. Despite the initial advantage of DCA-Na salt during the gastric phase, DCA-ISNT NCC achieved the highest overall dissolution after gastric to intestinal transfer, consistent with the superior in vitro dissolution performance.

**Figure 7:**
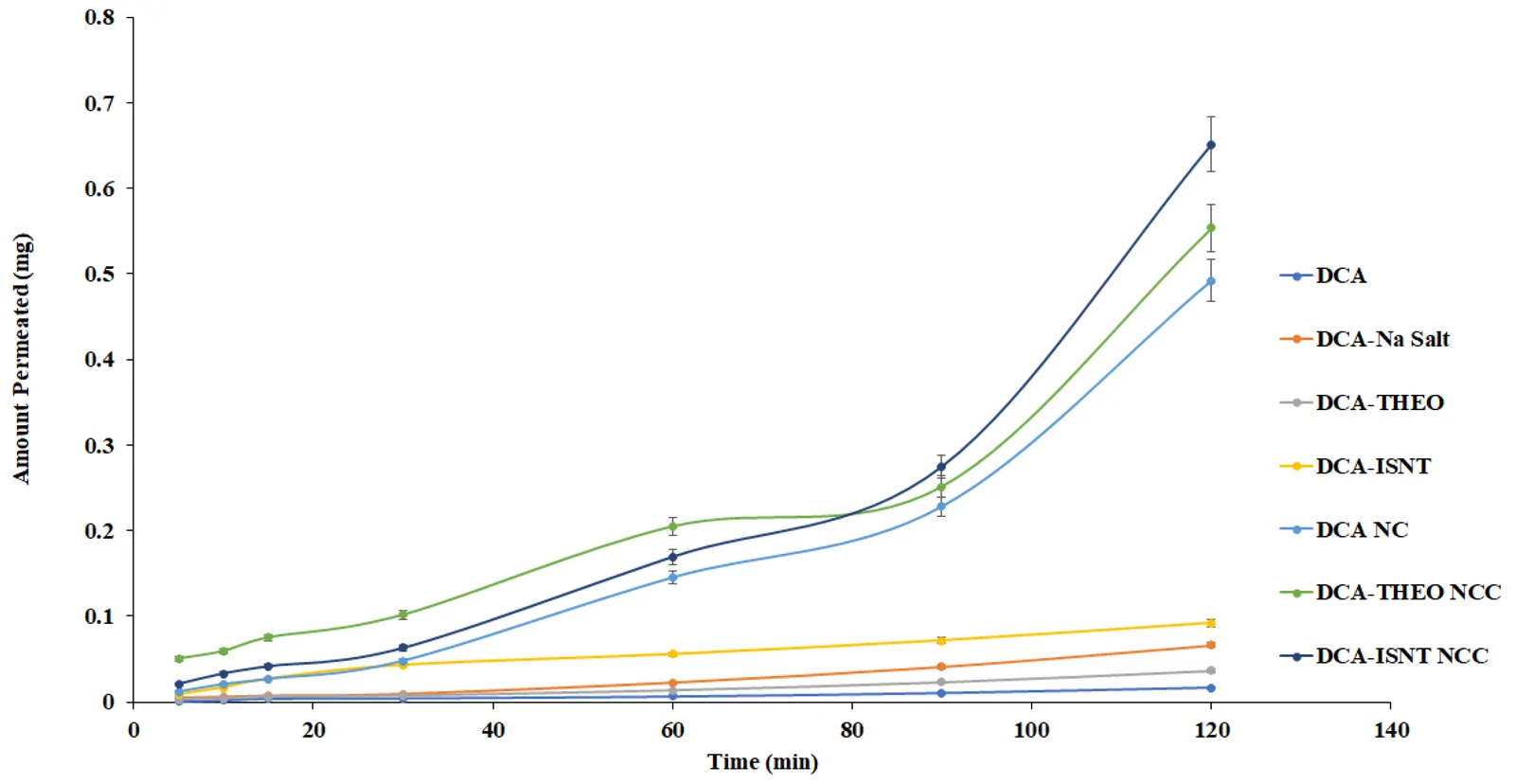
Uptake of DCA in the permeation chamber during flux study (n = 3).

The enhanced drug release observed during the gastric transfer study was reflected in the flux experiment outcomes. DCA-ISNT NCC exhibited the highest flux (1.43 ± 0.071 μg/cm^2^·min), followed by DCA-THEO NCC and DCA-NC, whereas DCA exhibited the lowest flux (0.04 ± 0.002 μg/cm^2^·min). The results of the flux experiment are presented in Figure 7 and Table 6. The higher flux of the nanococrystals can be attributed to their enhanced drug release resulting from the combined effects of particle size reduction and crystal engineering. While particle size reduction facilitates rapid dissolution by decreasing the effective diffusion boundary layer surrounding the particles, the reduced lattice stability and improved wettability further promote drug dissolution. These factors generate higher dissolved drug concentrations in the donor compartment, enhancing transmembrane flux.

### 3.5. Pharmacokinetics of DCA After Oral Administration in Rats

**Figure 8:**
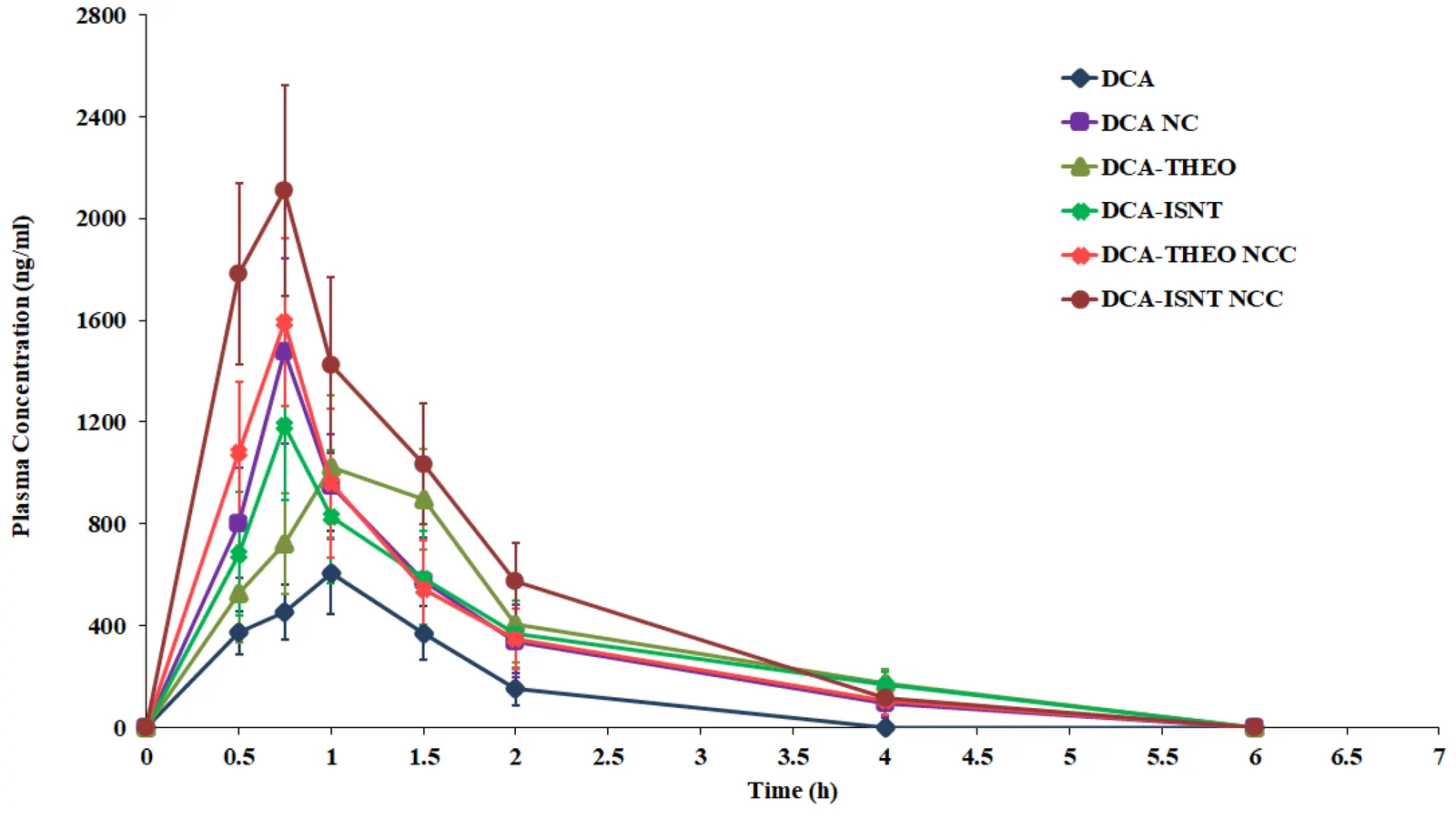
Plasma concentration–time of DCA after oral administration of DCA, and cocrystals and nanococrystals of DCA (n = 6).

The plasma concentration profiles of DCA and its formulations following oral administration in rats are presented in Figure 8, while the pharmacokinetic parameters are summarized in Table 7. Among the unmilled samples, DCA-THEO exhibited the longest T_max_ (1.11 ± 0.21 h), whereas DCA and DCA-ISNT showed comparable T_max_ values. After nanonization, all formulations exhibited shorter T_max_ values (0.56–0.57 h), indicating more rapid absorption than their unmilled counterparts. DCA-ISNT NCC exhibited the highest C_max_ (1927.20 ± 155.75 ng/mL), followed by DCA-THEO NCC (1418.89 ± 245.02 ng/mL) and DCA-NC (1312.61 ± 672.67 ng/mL). Similarly, DCA-ISNT NCC achieved the highest systemic exposure, with an AUC_0–∞_ of 3062.65 ± 526.91 ng/mL·h, showing a nearly three-fold increase compared with DCA. AUC_0–∞_ values of DCA NC and DCA-THEO NCC were comparable (2352.53 ± 537.78 and 2222.96 ± 151.19 ng/mL·h, respectively), while the unmilled cocrystals exhibited only a moderate improvement over the pure drug.

The improved pharmacokinetic profile of DCA-ISNT NCC is in good agreement with the dissolution, gastric transfer, and flux studies, demonstrating that the enhanced in vitro performance translated into improved in vivo oral exposure.

**Figure 9:**
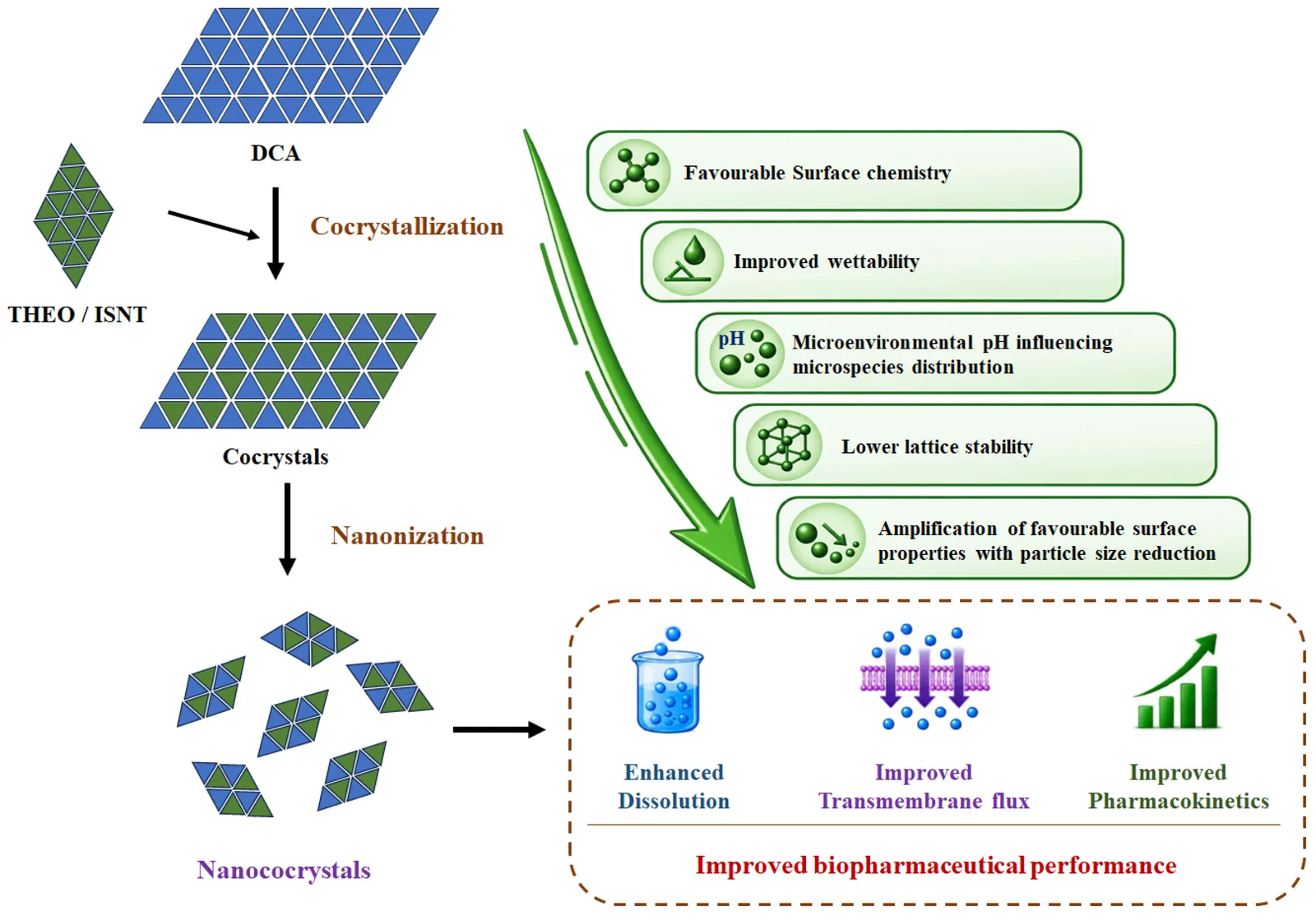
Schematic representation of the proposed mechanism of enhanced biopharmaceutical performance of DCA nanococrystals.

The results obtained in the present study indicate an interplay between crystal and particle characteristics in determining the performance of the nanococrystals. The proposed contribution of these interacting factors to the enhanced biopharmaceutical performance is schematically presented in Figure 9.

## 4. Conclusions

This study investigated the impact of nanococrystallization on the biopharmaceutical performance of diclofenac acid using theophylline- and isonicotinamide-based cocrystal systems. The results demonstrate that nanococrystallization offers an effective approach for improving the biopharmaceutical performance of poorly water-soluble drugs by integrating the benefits of crystal engineering and particle engineering. The findings indicate that particle size reduction alone does not fully account for the observed performance of nanococrystals and highlight the importance of considering crystal properties alongside particle characteristics during formulation development. Future studies involving a broader range of drug-coformer systems and storage stability evaluations will help to further understand the applicability of this formulation strategy across different pharmaceutical systems.

## Figures and Tables

**Table 1:** Dissolution efficiency (DE, %) of DCA, DCA-THEO, DCA-ISNT, DCA-NC, DCA-THEO NCC, and DCA-ISNT NCC calculated over 0–30, 0–60, and 0–120 min in pH 1.2, pH 4.5, and pH 6.8 dissolution media.

System	pH 1.2			pH 4.5			pH 6.8		
	DE_0–30_	DE_0–60_	DE_0–120_	DE_0–30_	DE_0–60_	DE_0–120_	DE_0–30_	DE_0–60_	DE_0–120_
DCA	2.4	3.4	6.0	13.3	16.7	22.1	62.5	70.5	76.7
DCA-THEO	5.9	8.2	12.5	20.3	30.2	37.8	66.1	76.1	83.0
DCA-ISNT	6.7	10.6	15.2	35.6	39.7	44.3	75.9	81.7	87.9
DCA-NC	4.9	8.8	12.5	28.1	34.7	39.0	77.0	83.2	89.8
DCA-THEO NCC	8.8	14.6	18.5	29.8	43.0	48.2	72.0	81.0	88.4
DCA-ISNT NCC	17.6	22.8	22.5	56.6	60.5	58.7	80.4	87.6	92.8

DE is calculated from the mean dissolution profiles using the trapezoidal method according to Khan and Rhodes.

**Table 2:** Surface-exposed functionalities on major facets of DCA, DCA-THEO, and DCA-ISNT.

System	Facet	% Facet Area	Predominantly Exposed Hydrophilic Functionalities
DCA	2 0 0	27.56	-
	2 0 −2	22.50	-
	0 0 2	21.19	-
	1 1 0	17.01	Carboxylic acid (-COOH, DCA)
DCA-THEO	0 0 1	41.58	Lactam carbonyl (-C=O, THEO)
	0 1 0	21.70	Secondary amine (-NH-, DCA)
	0 1 1	15.88	Lactam carbonyl (-C=O, THEO)
DCA-ISNT	0 0 1	58.88	-
	0 1 0	20.45	Amide (-CONH_2_, ISNT) and carboxylic acid (-COOH, DCA)
	1 0 0	8.10	-Carboxylic acid (-COOH, DCA) and pyridine nitrogen (ISNT)

**Table 3:** Microenvironmental pH and % distribution of MS II of DCA for studied systems in pH 1.2 and pH 4.5 aqueous buffers.

System	Microenvironmental pH		% Distribution of MS II of DCA	
	pH 1.2	pH 4.5	pH 1.2	pH 4.5
DCA	1.28	4.55	99.4	22.4
DCA-THEO	1.72	4.56	99.3	22.4
DCA-ISNT	3.81	4.91	61.4	11.3

**Table 4:** Lattice energies and contribution of individual components for DCA, DCA-THEO, and DCA-ISNT systems.

System	Lattice Energy (kcal/mol)	vdW Contribution (kcal/mol)	Electrostatic Contribution (kcal/mol)
DCA	−283.864	−216.486	−67.378
DCA-THEO	−135.789	−96.666	−39.123
DCA-ISNT	−126.107	−76.985	−49.123

**Table 5:** Strongest intermolecular pair interaction energies of DCA, DCA-THEO and DCA-ISNT systems. E_ele_, E_dis_, and E_tot_ represent electrostatic contribution, dispersion contribution, and total interaction energy, respectively.

System	Molecular Pair	Centroid Distance, R (Å)	*E_ele_* (kJ/mol)	*E_dis_* (kJ/mol)	*E_tot_* (kJ/mol)
DCA	DCA···DCA	9.33	−120.8	−15.7	−73.4
DCA-THEO	DCA···THEO	6.94	−11.3	−39.5	−30.9
	DCA···DCA	8.21	−4.4	−34.2	−21.0
DCA-ISNT	DCA···ISNT	7.84	−5.2	−29.3	−20.0
	DCA···DCA	7.46	−5.2	−29.3	−20.0

**Table 6:** Flux rate of all the studied systems (n = 3).

Name	Flux (µg/cm^2^·min)
DCA	0.04 ± 0.002
DCA-Na Salt	0.15 ± 0.007
DCA-THEO	0.08 ± 0.004
DCA-ISNT	0.20 ± 0.010
DCA-NC	1.08 ± 0.054
DCA-THEO NCC	1.21 ± 0.061
DCA-ISNT NCC	1.43 ± 0.071

**Table 7:** PK parameters following the oral administration of DCA, cocrystals, nanocrystals, and nanococrystal suspensions (n = 6).

System	t_max_ (h)	C_max_ (ng/mL)	AUC_0–∞_ (ng/mL·h)
DCA	0.77 ± 0.17 ^ab^	493.53 ± 53.58 ^b^	1019.30 ± 97.72 ^a^
DCA-NC	0.57 ± 0.27 ^b^	1312.61 ± 672.67 ^cd^	2352.53 ± 537.78 ^bc^
DCA-THEO	1.11 ± 0.21 ^a^	833.36 ± 184.56 ^bd^	2484.44 ± 372.22 ^bc^
DCA-ISNT	0.53 ± 0.29 ^b^	983.08 ± 160.70 ^bc^	2184.60 ± 541.98 ^b^
DCA-THEO NCC	0.56 ± 0.24 ^b^	1418.89 ± 245.02 ^ac^	2222.96 ± 151.19 ^b^
DCA-ISNT NCC	0.57 ± 0.16 ^b^	1927.20 ± 155.75 ^a^	3062.65 ± 526.91 ^c^

Different superscript letters indicate statistically significant differences between groups (*p* < 0.05).

## Data Availability

The original contributions presented in this study are included in the article/Supplementary Material. Further inquiries can be directed to the corresponding author.

## Supplementary Materials

The following supporting information can be downloaded at: https://www.mdpi.com/article/10.3390/pharmaceutics18091119/s1.
