# Supplementary material for: Nanococrystals of Diclofenac Acid to Improve Biopharmaceutical Performance: Understanding the Key Drivers
Source: Pharmaceutics. 2026 Sep 6;18(9):1119. doi: 10.3390/pharmaceutics18091119 (PMC13610591; doi:10.3390/pharmaceutics18091119)
Supplement: Supplementary file 1 [file pharmaceutics-18-01119-s001.zip › pharmaceutics-4498855-supplementary.pdf]

Tables

Table S1. Description of process parameters for generation of DCA-THEO NCC, DCA-ISNT NCC, and DCA-NC 3
Table S2. Screening of stabilizers and optimization of various process parameters during generation of nanococrystal and nanocrystals 4
Table S3. Particle size of DCA, DCA-THEO NCC, and DCA-ISNT NCC by DLS (n=3) 5
Table S4. Surface elemental composition of studied systems determined by XPS 6
Table S5. Major facets, d-spacing and their attachment energies for the studied systems 6
Table S6. Interaction energies for DCA 7
Table S7. Interaction energies for DCA-THEO 7
Table S8. Interaction energies for DCA-ISNT 8

Figures

Figure S1. Schematic representation of the in-house apparatus used for the in vitro flux study. The figure was created using an AI-assisted illustration tool and subsequently edited for accuracy and labelling. 7
Figure S2. PLM images of (A) DCA, (B) THEO, and (C) ISNT 8
Figure S3. DSC heating curves of (A) DCA, (B) THEO, and (C) ISNT 9
Figure S4. PLM images of (A) DCA-THEO and (B) DCA-ISNT 10
Figure S5. DSC heating curves of (A) DCA-THEO and (B) DCA-ISNT 10
Figure S6. Overlay of simulated and experimental PXRD patterns of (A) DCA-THEO and (B) DCA-ISNT 11
Figure S7. DSC heating curves of (A) DCA-THEO and (B) DCA-ISNT before and after milling 12
Figure S8. Comparison of FTIR spectra of (A) DCA, (B) DCA-THEO, and (C) DCA-ISNT before and after milling 13
Figure S9. Surface exposed polar functionalities on major facets of DCA, DCA-THEO, and DCA-ISNT 14
Figure S10. MLSP mapping of (A) DCA, (B) THEO, and (C) ISNT 15
Figure S11. Visualization of proposed cleavage plane in the supercell structures of DCA, DCA-THEO, and DCA-ISNT 16
Figure S12. Percentage microspecies distribution of THEO over the pH range 0-7 16

Figure S13. Percentage microspecies distribution of ISNT over the pH range 0-7 17

Figure S14. Energy framework of DCA viewed along different crystallographic axes......... 17

Figure S15. Energy framework of DCA-THEO viewed along different crystallographic axes 18

Figure S16. Energy framework of DCA-ISNT viewed along different crystallographic axes 19

Table S1. *Description of process parameters for generation of DCA-THEO NCC, DCA-ISNT NCC, and DCA-NC*

**Parameters**	**Description**
Cocrystal & Drug amount	The drug amount was varied from 5 to 15 % in a set of experiments from batch D6 to D8
Amount of stabilizer	The amount of stabilizer (SLS+HPC-EF) was varied in a set of experiments from batch D9 to D12. The effect of the amount of stabilizer on the particle size of DCA-THEO nanococrystal, DCA-ISNT nanococrystal and DCA nanocrystals was evaluated by PSD and PDI analysis
Stirring speed	The stirring speed was varied from 500 to 1500 rpm in a set of experiments from batch D13 to D15
Stirring time	The impact of stirring time was evaluated in a set of experiments from batch D1 to D5. The particle size and PDI of generated nanocrystals were performed at the different stirring times such as 1 h, 2 h, 4 h, and 6 h
Size of bead	The impact of the size of glass beads was evaluated in a set of experiments from batch D16 to D19. Four different sizes of glass beads such as 0.1-0.2 mm, 0.2-0.3 mm, 0.3-0.4 mm and 0.5-0.7 mm were selected for the present experiments
Amount of beads	The amount of glass beads was varied from 2 to 8 g in a set of experiments from batch D20 to D23
Volume of medium	The volume of medium was varied from 3 to 7 ml in a set of experiments from batch D24 to D26

Table S2. *Screening of stabilizers and optimization of various process parameters during generation of nanococrystal and nanocrystals*

Batch No.	Stabilizers	Drug amount (%)	Amount of stabilizer (%)	Stirring speed (rpm)	Stirring time (h)	Bead size (mm)	Bead amount (g)	Volume of medium (ml)
**Stabilizer type and stirring time**								
D1	SLS+HPC-EF	5	0.1+1	1500	1, 2, 4, 6,	0.2-0.3	8	3
D2	SLS	5	0.1	1500	1, 2, 4, 6,	0.2-0.3	8	3
D3	HPC-EF	5	1	1500	1, 2, 4, 6,	0.2-0.3	8	3
D4	Ploloxmer188+HPC-EF	5	0.1+1	1500	1, 2, 4, 6,	0.2-0.3	8	3
D5	Poloxamer 188	5	0.1	1500	1, 2, 4, 6,	0.2-0.3	8	3
**Cocrystal & Drug amount**								
D6	SLS+HPC-EF	5	0.1+1	1500	6	0.2-0.3	8	3
D7	SLS+HPC-EF	10	0.1+1	1500	6	0.2-0.3	8	3
D8	SLS+HPC-EF	15	0.1+1	1500	6	0.2-0.3	8	3
**Amount of stabilizer (SLS+HPC-EF)**								
D9	SLS+HPC-EF	5	0.5+0.5	1500	6	0.2-0.3	8	3
D10	SLS+HPC-EF	5	0.2+1	1500	6	0.2-0.3	8	3
D11	SLS+HPC-EF	5	0.1+1	1500	6	0.2-0.3	8	3
D12	SLS+HPC-EF	5	0.1+1.5	1500	6	0.2-0.3	8	3
**Stirring speed**								
D13	SLS+HPC-EF	5	0.1+1	500	6	0.2-0.3	8	3

D14	SLS+HPC-EF	5	0.1+1	1000	6	0.2-0.3	8	3
D15	SLS+HPC-EF	5	0.1+1	1500	6	0.2-0.3	8	3
**Bead size**								
D16	SLS+HPC-EF	5	0.1+1	1500	6	0.1-0.2	8	3
D17	SLS+HPC-EF	5	0.1+1	1500	6	0.2-0.3	8	3
D18	SLS+HPC-EF	5	0.1+1	1500	6	0.3-0.4	8	3
D19	SLS+HPC-EF	5	0.1+1	1500	6	0.5-0.7	8	3
**Bead amount**								
D20	SLS+HPC-EF	5	0.1+1	1500	6	0.2-0.3	2	3
D21	SLS+HPC-EF	5	0.1+1	1500	6	0.2-0.3	4	3
D22	SLS+HPC-EF	5	0.1+1	1500	6	0.2-0.3	6	3
D23	SLS+HPC-EF	5	0.1+1	1500	6	0.2-0.3	8	3
**Volume of medium**								
D24	SLS+HPC-EF	5	0.1+1	1500	6	0.2-0.3	8	3
D25	SLS+HPC-EF	5	0.1+1	1500	6	0.2-0.3	8	5
D26	SLS+HPC-EF	5	0.1+1	1500	6	0.2-0.3	8	7

Table S3. *Particle size of DCA, DCA-THEO NCC, and DCA-ISNT NCC by DLS (n=3)*

**System**	**Z_{avg} (nm)**	**PDI**
DCA NC	311.2 ± 42.3	0.255 ± 0.023

DCA-THEO NCC	235.4 ± 65.4	0.190 ± 0.041
DCA-ISNT NCC	698.0 ± 90.2	0.213 ± 0.034

Table S4. *Surface elemental composition of studied systems determined by XPS*

Atoms	DCA	DCA-THEO	DCA-ISNT	DCA-NC	DCA-THEO NCC	DCA- ISNT NCC
Cl	10.6	6.3	7.5	9.5	6.4	7.1
C	73.5	69.6	72.6	71.3	67.5	71.3
N	5.2	13.2	9.3	5.9	12.5	8.5
O	10.5	11.3	10.6	13.2	13.5	13.1

Table S5. *Major facets, d-spacing and their attachment energies for the studied systems*

System	Facet	% Facet area	dhkl (Å)	Attachment energy (kcal/mol)	Proposed cleavage plane
DCA	2 0 0	27.56	9.55	-93.90192191	2 0 0
	2 0 -2	22.50	8.25	-96.76919314	
	0 0 2	21.19	9.47	-107.28923408	
	1 1 0	17.01	6.56	-160.97105295	
DCA-THEO	0 0 1	41.58	11.63	-25.51873851	0 0 1
	0 1 0	21.70	11.55	-39.10912154	
	0 1 1	15.88	9.55	-37.50997807	
DCA-ISNT	0 0 1	58.88	14.78	-19.31018278	0 0 1
	0 1 0	20.45	8.75	-48.58927441	
	1 0 0	8.10	7.39	-67.01941204	

Table S6. *Interaction energies for DCA*

	N	Symop	R	Electron Density	E_ele	E_pol	E_dis	E_rep	E_tot
	2	-x, y, -z+1/2	9.19	B3LYP/6-31G(d,p)	-0.6	-0.3	-7.5	2.0	-6.2
	1	-x, y, -z+1/2	5.97	B3LYP/6-31G(d,p)	-9.4	-1.1	-49.2	34.4	-32.4
	1	-x, -y, -z	9.51	B3LYP/6-31G(d,p)	-4.7	-0.6	-16.7	11.5	-12.9
	2	x+1/2, y+1/2, z	10.73	B3LYP/6-31G(d,p)	-1.0	-0.4	-8.7	3.4	-6.8
	1	-x, -y, -z	9.76	B3LYP/6-31G(d,p)	-0.1	-0.2	-4.3	3.8	-1.6
	2	x, y, z	7.00	B3LYP/6-31G(d,p)	-2.3	-1.6	-25.2	14.8	-16.3
	2	-x+1/2, y+1/2, -z+1/2	8.31	B3LYP/6-31G(d,p)	-4.6	-0.9	-26.9	16.9	-18.6
	1	-x+1/2, -y+1/2, -z	5.77	B3LYP/6-31G(d,p)	-6.1	-1.6	-40.4	15.4	-33.3
	1	-x+1/2, -y+1/2, -z	9.33	B3LYP/6-31G(d,p)	-120.8	-26.9	-15.7	142.3	-73.4

Table S7. *Interaction energies for DCA-THEO*

	N	Symop	R	Electron Density	E_ele	E_pol	E_dis	E_rep	E_tot
	1	-x, -y, -z	8.21	B3LYP/6-31G(d,p)	-4.4	-0.9	-34.2	22.7	-21.0
	2	x, y, z	7.53	B3LYP/6-31G(d,p)	-3.2	-1.0	-21.7	10.2	-16.7
	1	-	9.37	B3LYP/6-31G(d,p)	0.6	-0.0	-0.6	0.0	0.1
	1	-x, -y, -z	6.94	B3LYP/6-31G(d,p)	-11.3	-1.5	-39.5	26.9	-30.9
	1	-	8.45	B3LYP/6-31G(d,p)	-0.2	-0.0	-0.4	0.0	-0.5
	1	-	8.17	B3LYP/6-31G(d,p)	0.5	-0.0	-0.5	0.0	0.1
	1	-x, -y, -z	6.45	B3LYP/6-31G(d,p)	-5.8	-0.6	-22.8	15.4	-16.9
	1	-	7.05	B3LYP/6-31G(d,p)	-0.0	-0.0	-1.3	0.0	-1.2
	1	-	7.59	B3LYP/6-31G(d,p)	-3.2	-1.0	-21.7	10.2	-16.7
	1	-x, -y, -z	10.27	B3LYP/6-31G(d,p)	-8.2	-3.6	-14.7	14.2	-15.3

Table S8. *Interaction energies for DCA-ISNT*

	N	Symop	R	Electron Density	E_ele	E_pol	E_dis	E_rep	E_tot
	2	x, y, z	7.46	B3LYP/6-31G(d,p)	-5.2	-1.7	-29.3	19.9	-20.0
	1	-x, -y, -z	9.44	B3LYP/6-31G(d,p)	-1.1	-0.3	-13.8	4.7	-10.4
	2	x, y, z	10.82	B3LYP/6-31G(d,p)	-2.0	-0.6	-14.0	11.4	-7.7
	1	-x, -y, -z	7.83	B3LYP/6-31G(d,p)	-7.6	-1.4	-28.9	23.7	-19.5
	1	-	7.92	B3LYP/6-31G(d,p)	-2.0	-0.6	-14.0	11.4	-7.7
	1	-	6.63	B3LYP/6-31G(d,p)	-0.0	-0.2	-8.8	2.4	-6.3
	1	-	6.36	B3LYP/6-31G(d,p)	-0.2	-0.0	-1.1	0.0	-1.2
	2	x, y, z	8.82	B3LYP/6-31G(d,p)	-0.0	-0.2	-8.8	2.4	-6.3
	1	-x, -y, -z	6.57	B3LYP/6-31G(d,p)	-6.1	-0.7	-27.4	18.3	-19.5
	1	-	7.84	B3LYP/6-31G(d,p)	-5.2	-1.7	-29.3	19.9	-20.0
	1	-x, -y, -z	10.50	B3LYP/6-31G(d,p)	-1.3	-0.1	-6.1	1.3	-6.1

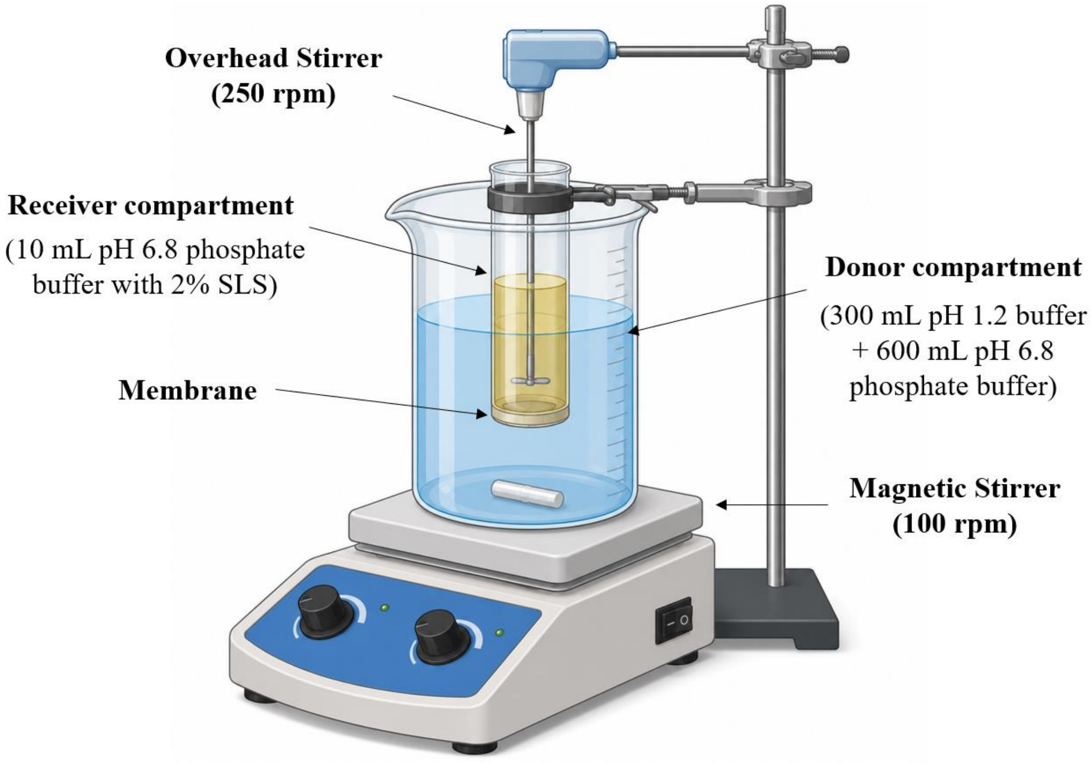

Figure S1. *Schematic representation of the in-house apparatus used for the in vitro flux study. The figure was created using an AI-assisted illustration tool and subsequently edited for accuracy and labelling.*

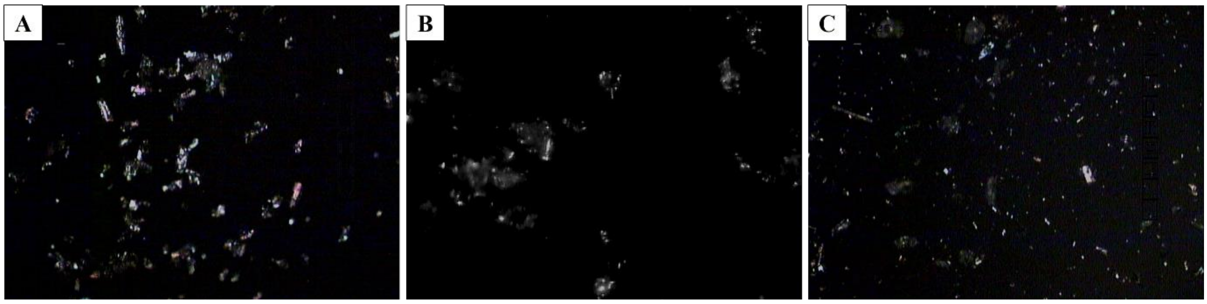

Figure S2. *PLM images of (A) DCA, (B) THEO, and (C) ISNT*

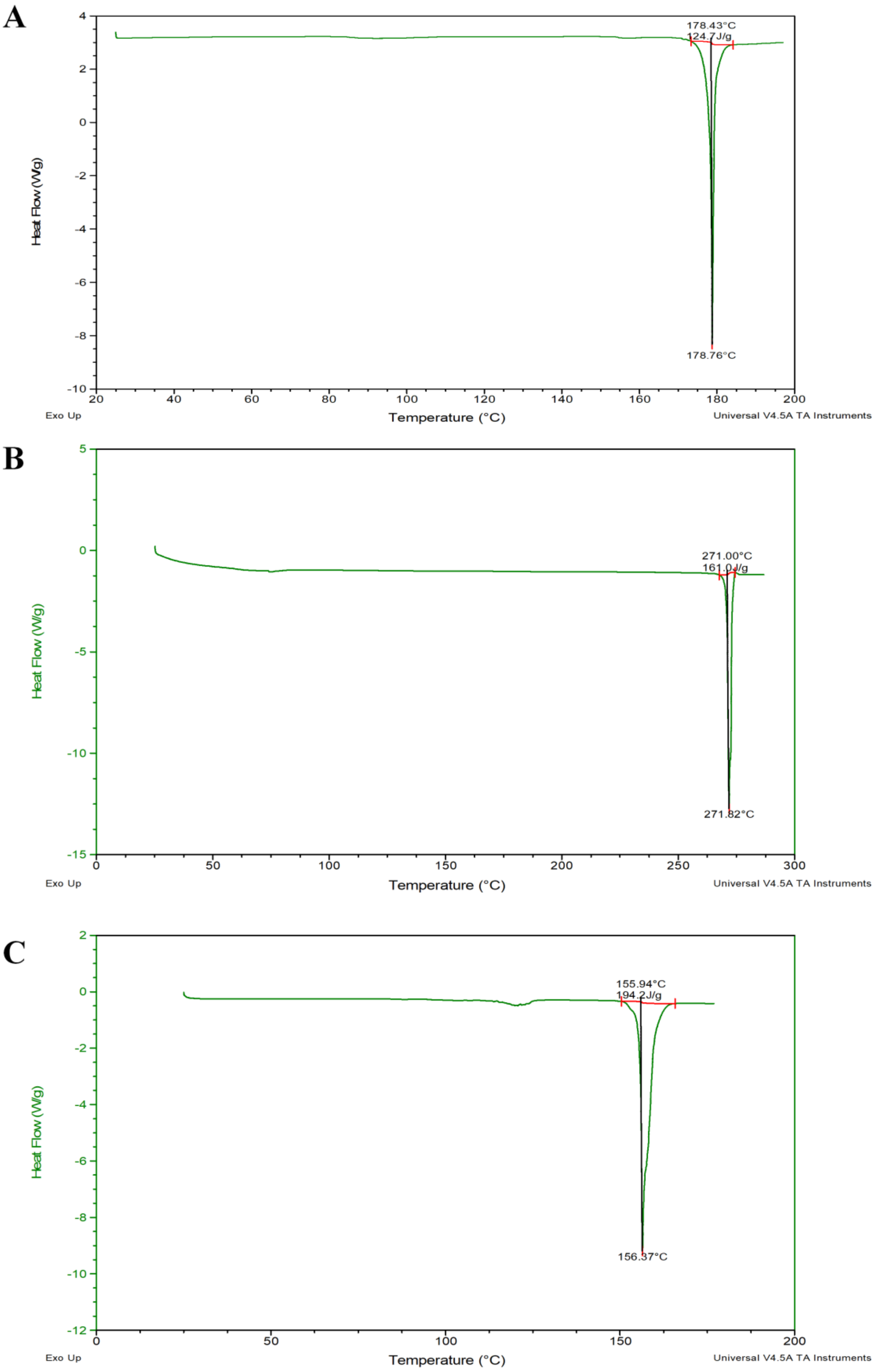

Figure S3. *DSC heating curves of (A) DCA, (B) THEO, and (C) ISNT*

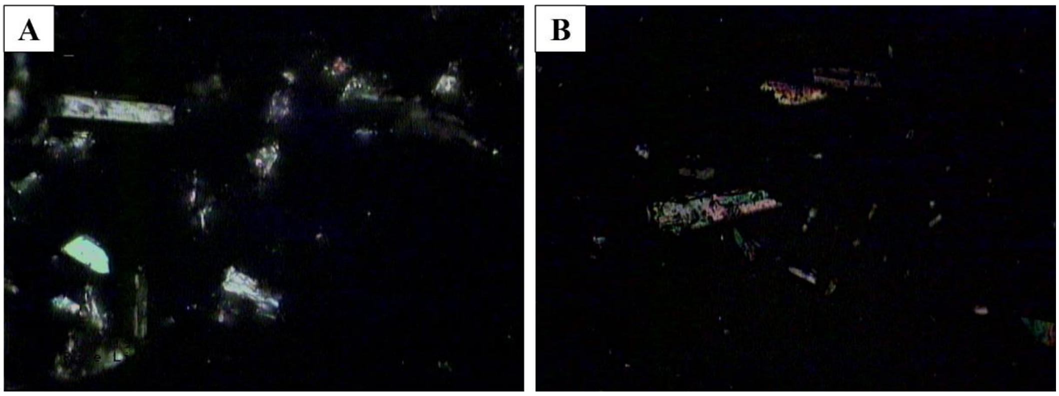

Figure S4. *PLM images of (A) DCA-THEO and (B) DCA-ISNT*

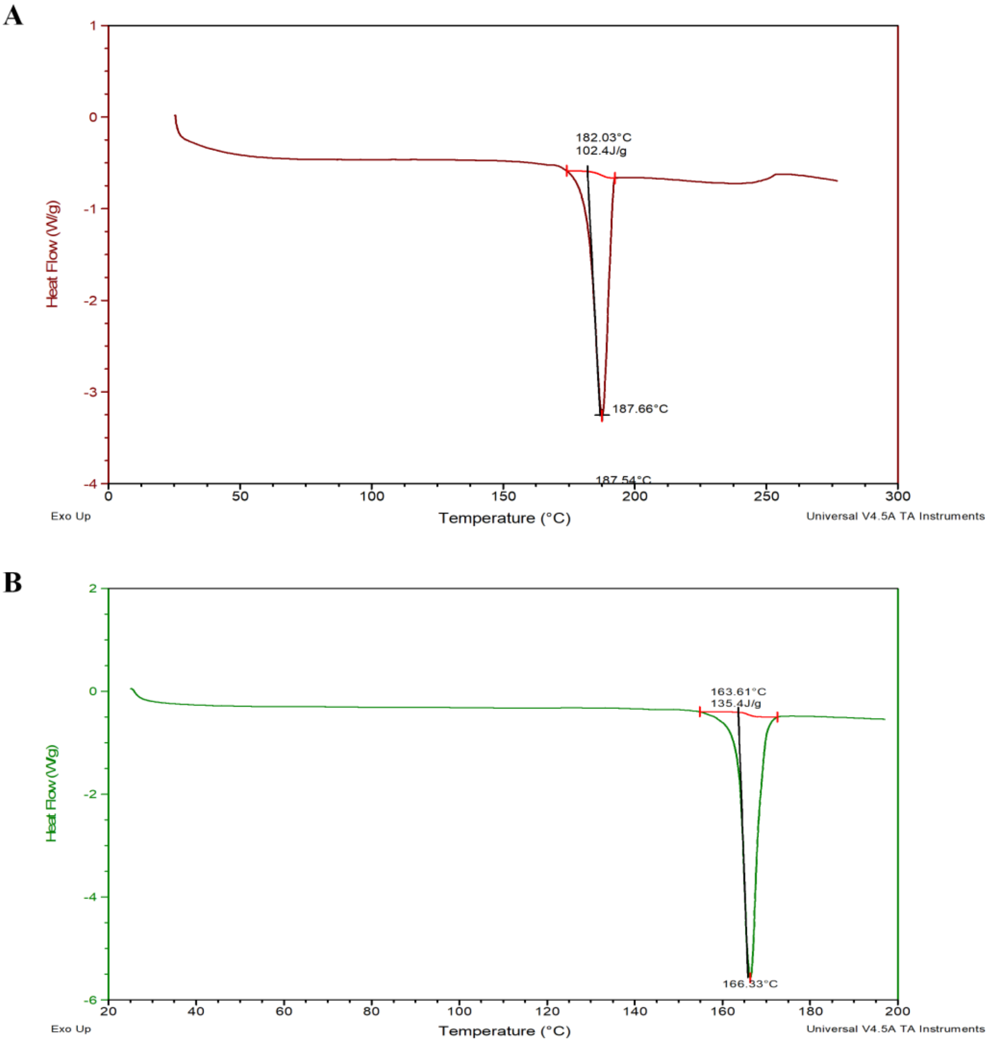

Figure S5. *DSC heating curves of (A) DCA-THEO and (B) DCA-ISNT*

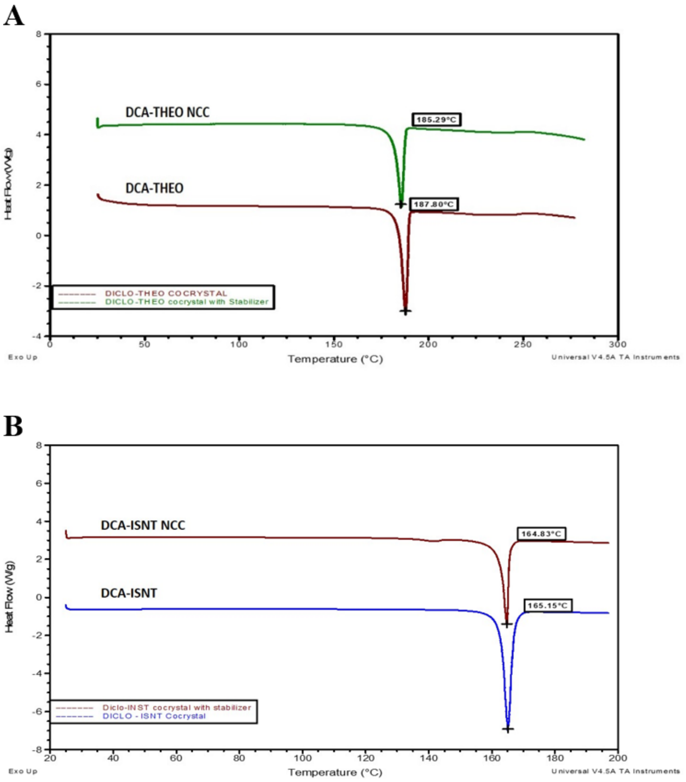

Figure S6. *DSC heating curves of (A) DCA-THEO and (B) DCA-ISNT before and after milling*

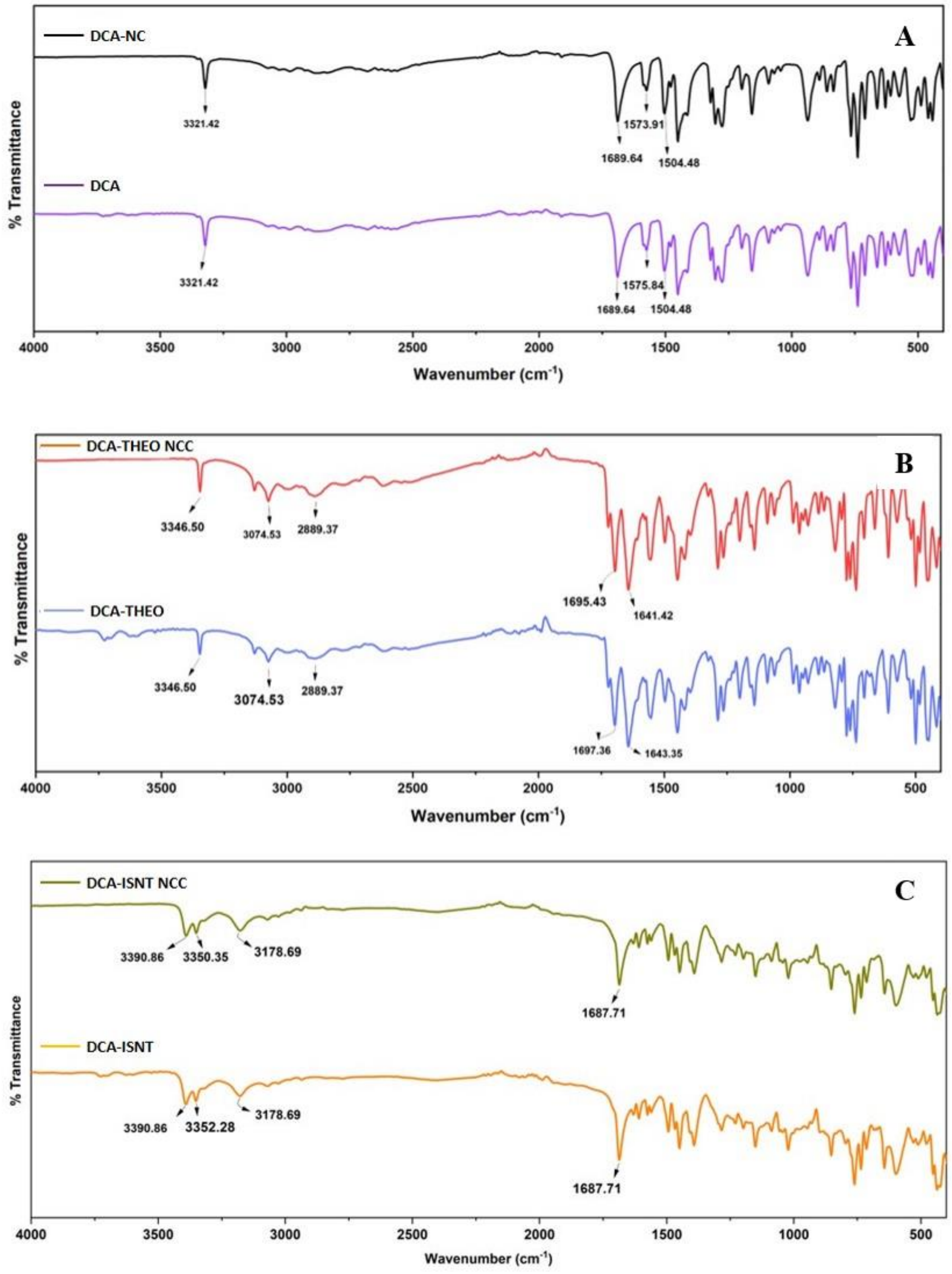

Figure S7. *Comparison of FTIR spectra of (A) DCA, (B) DCA-THEO, and (C) DCA-ISNT before and after milling*

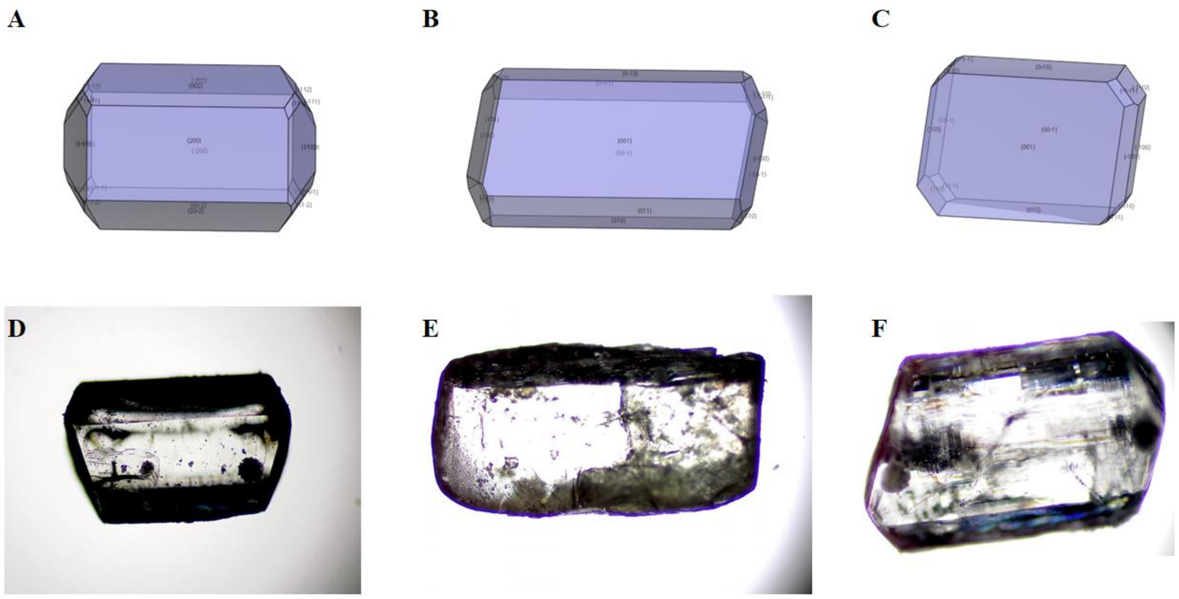

Figure S8. *Predicted growth morphologies and experimentally observed crystal habits of (A, D) DCA, (B, E) DCA-THEO, and (C, F) DCA-ISNT. A-C show the crystal morphologies predicted using the Growth Morphology module of Materials Studio, while D-F show the corresponding crystal obtained by solvent evaporation*

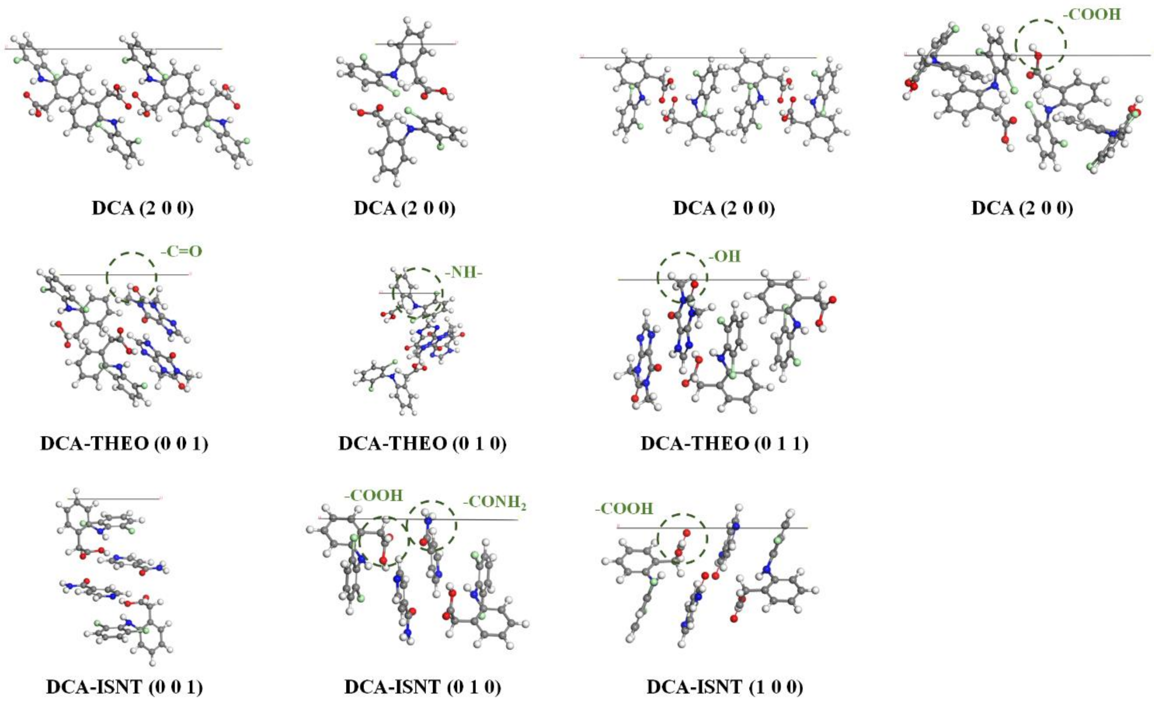

Figure S9. *Surface exposed polar functionalities on major facets of DCA, DCA-THEO, and DCA-ISNT*

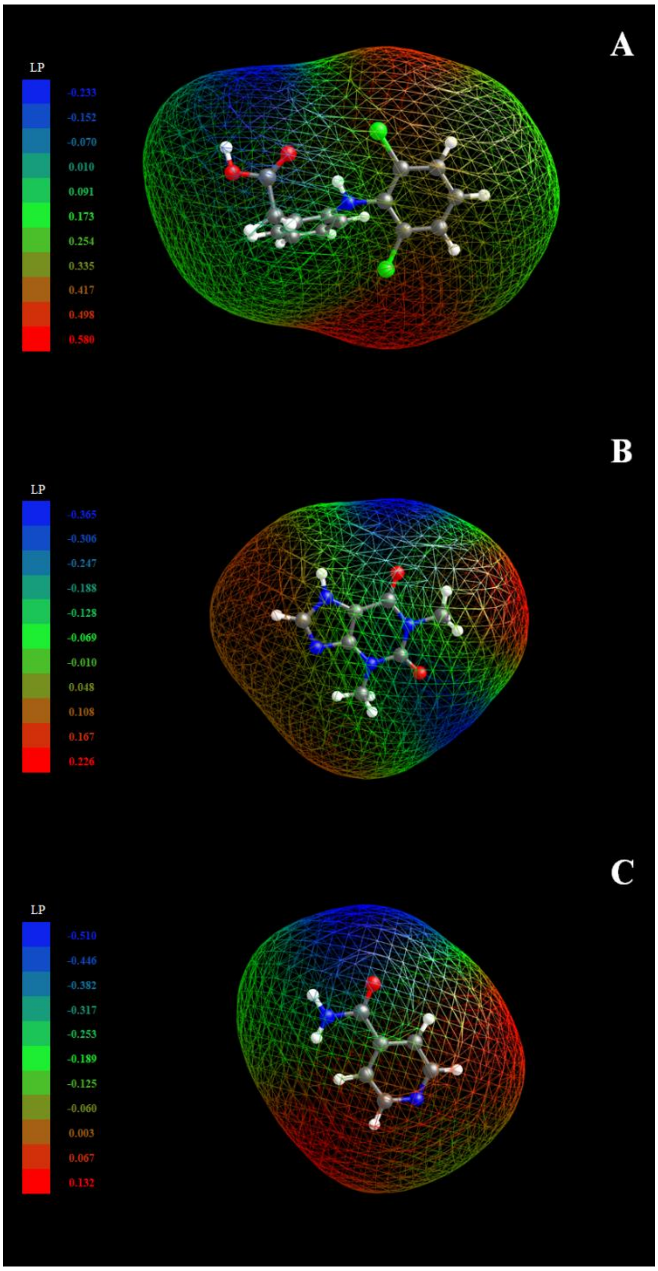

Figure S10. *MLSP mapping of (A) DCA, (B) THEO, and (C) ISNT*

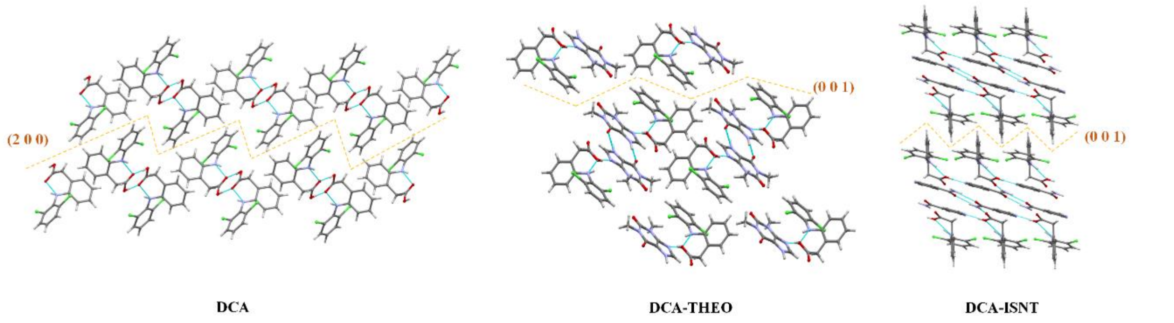

Figure S11. *Visualization of proposed cleavage plane in the supercell structures of DCA, DCA-THEO, and DCA-ISNT*

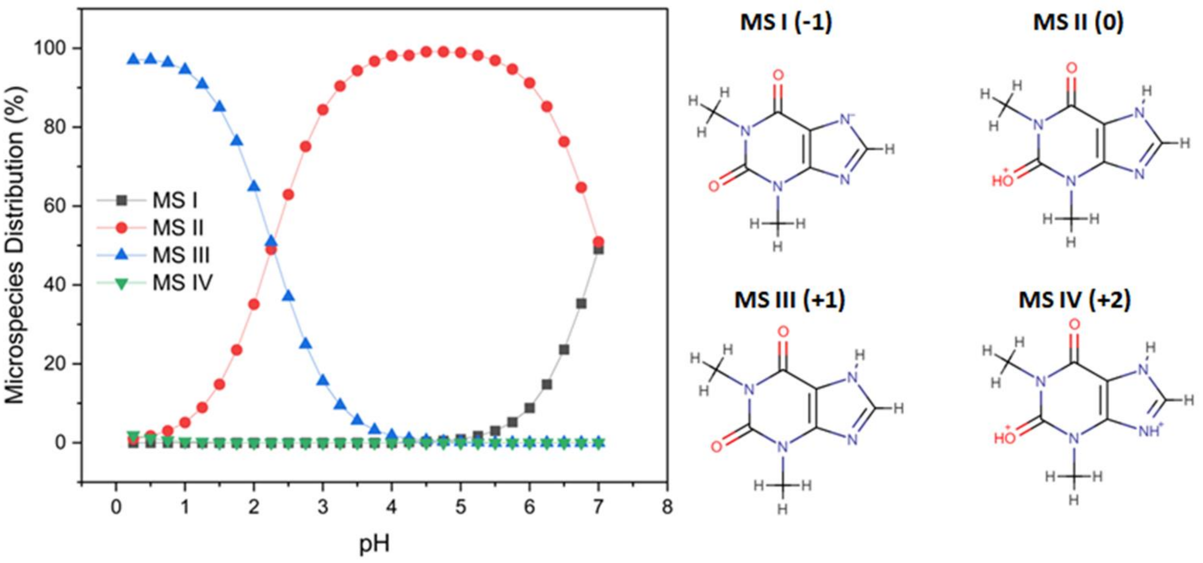

Figure S12. *Percentage microspecies distribution of THEO over the pH range 0-7*

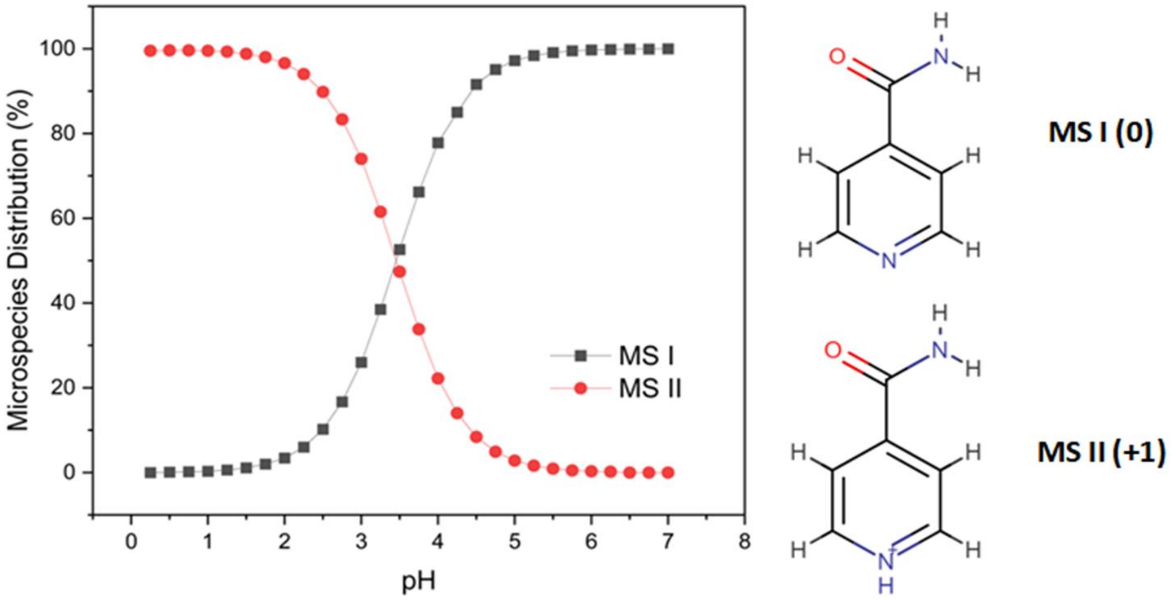

Figure S13. *Percentage microspecies distribution of ISNT over the pH range 0-7*

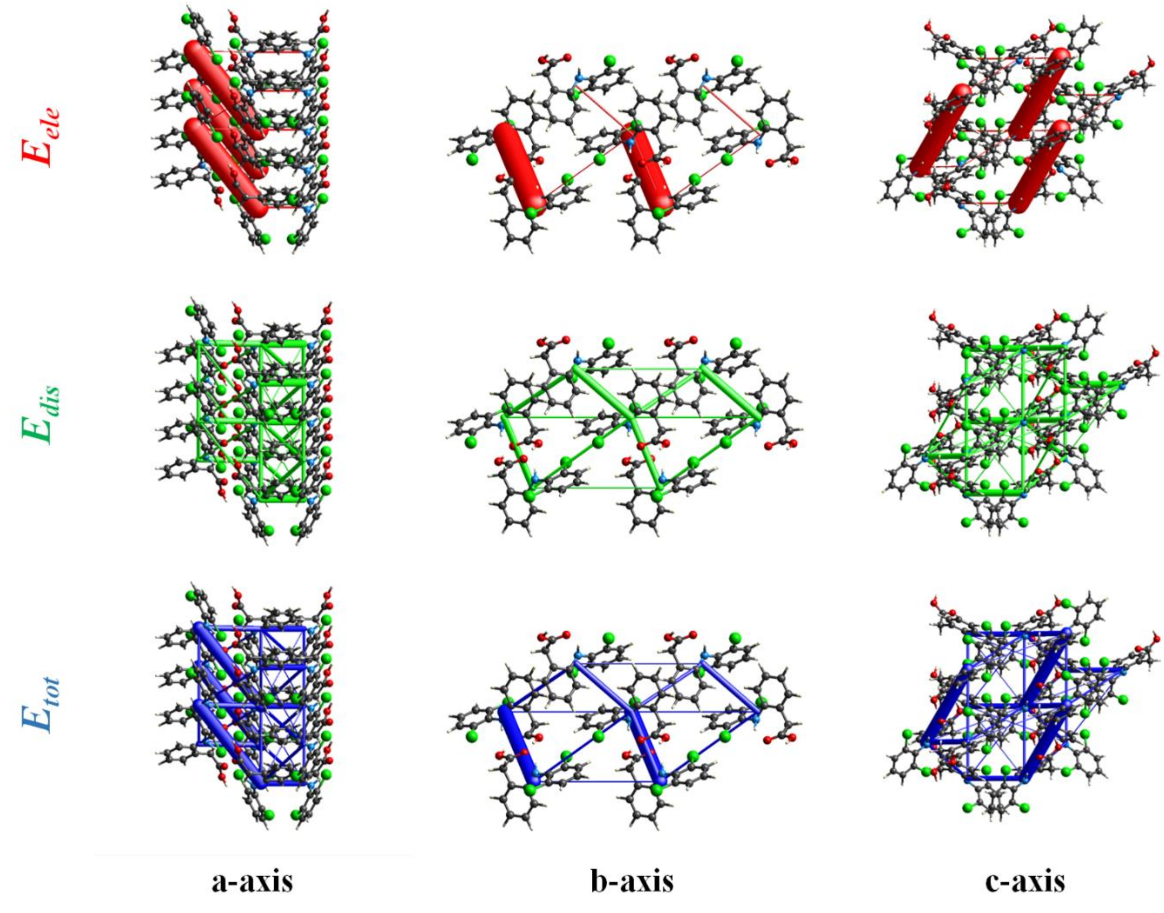

Figure S14. *Energy framework of DCA viewed along different crystallographic axes*

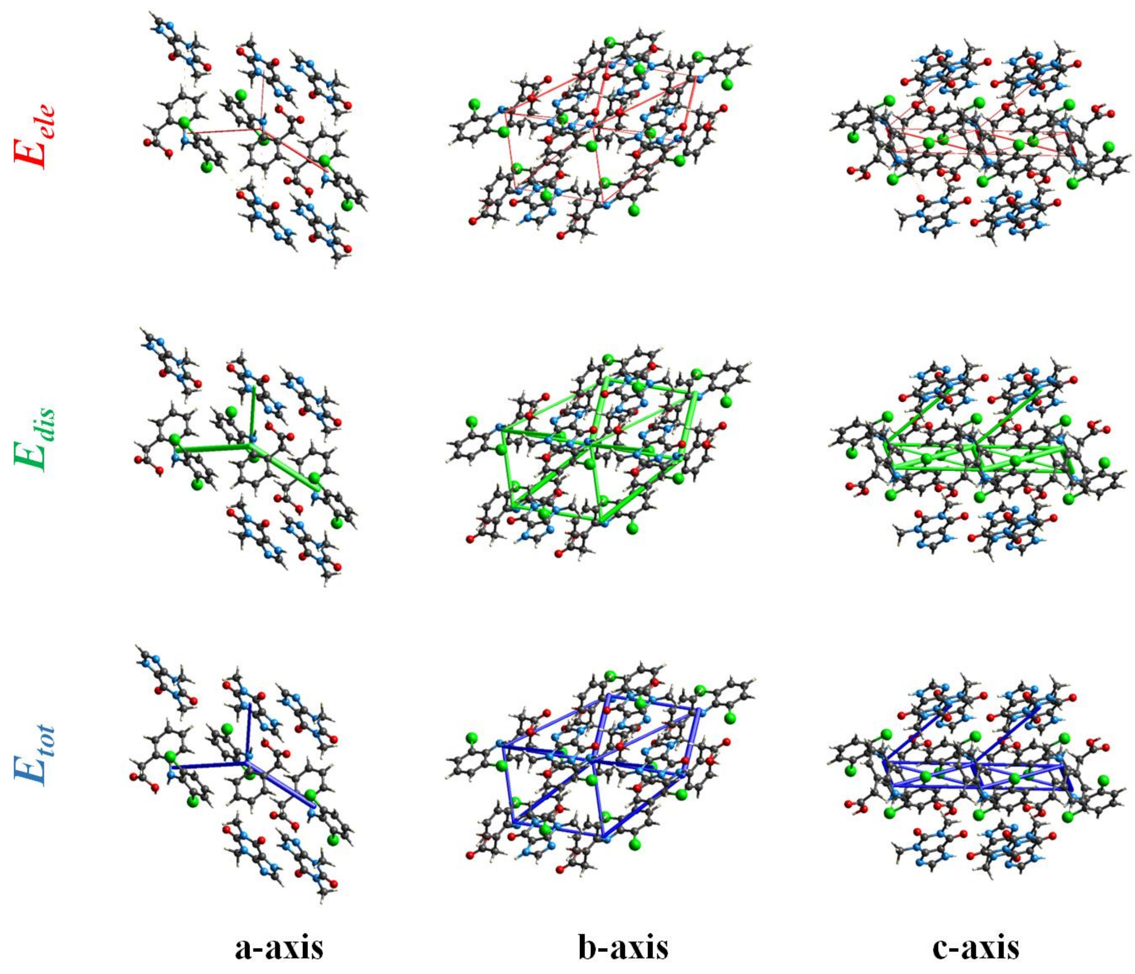

Figure S15. *Energy framework of DCA-THEO viewed along different crystallographic axes*

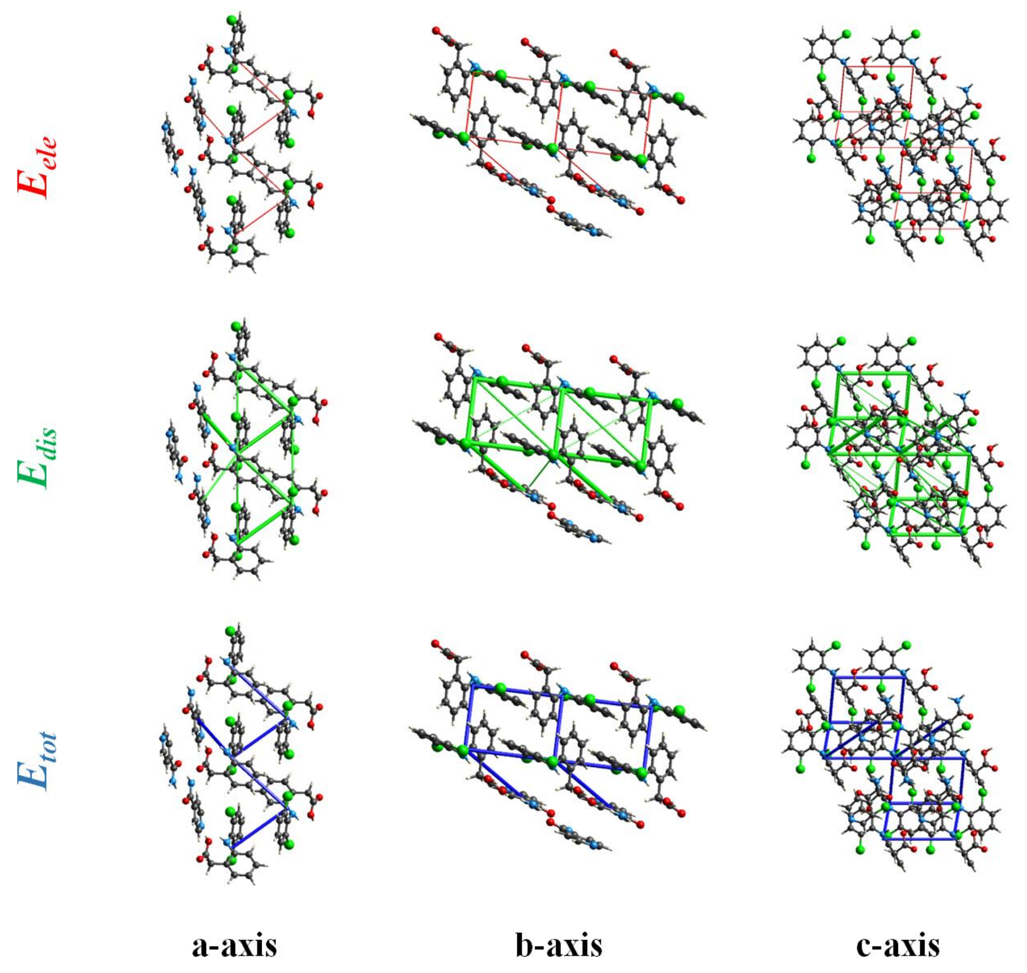

Figure S16. *Energy framework of DCA-ISNT viewed along different crystallographic axes*
